# Using machine learning for predicting intensive care unit resource use during the COVID-19 pandemic in Denmark

**DOI:** 10.1038/s41598-021-98617-1

**Published:** 2021-09-23

**Authors:** Stephan Sloth Lorenzen, Mads Nielsen, Espen Jimenez-Solem, Tonny Studsgaard Petersen, Anders Perner, Hans-Christian Thorsen-Meyer, Christian Igel, Martin Sillesen

**Affiliations:** 1 Department of Computer Science, University of Copenhagen, Copenhagen, Denmark; 2 Department of Clinical Pharmacology, Copenhagen University Hospital, Bispebjerg, Copenhagen, Denmark; 3 Department of Intensive Care, Copenhagen University Hospital, Rigshospitalet, Copenhagen, Denmark; 4 Department of Surgical Gastroenterology, Copenhagen University Hospital, Rigshospitalet, Copenhagen, Denmark; 5 Center for Surgical Translational and Artificial Intelligence Research (CSTAR), Copenhagen University Hospital, Rigshospitalet, Copenhagen, Denmark; 6 Present Address: Department of Clinical Medicine, University of Copenhagen, Copenhagen, Denmark; 7 Copenhagen Phase IV Unit (Phase4CPH), Department of Clinical Pharmacology, Center for Clinical Research and Prevention, Copenhagen University Hospital, Bispebjerg and Frederiksberg, Copenhagen, Denmark

**Keywords:** Computational biology and bioinformatics, Virology

## Abstract

The COVID-19 pandemic has put massive strains on hospitals, and tools to guide hospital planners in resource allocation during the ebbs and flows of the pandemic are urgently needed. We investigate whether machine learning (ML) can be used for predictions of intensive care requirements a fixed number of days into the future. Retrospective design where health Records from 42,526 SARS-CoV-2 positive patients in Denmark was extracted. Random Forest (RF) models were trained to predict risk of ICU admission and use of mechanical ventilation after *n* days (*n* = 1, 2, …, 15). An extended analysis was provided for *n* = 5 and *n* = 10. Models predicted *n*-day risk of ICU admission with an area under the receiver operator characteristic curve (ROC-AUC) between 0.981 and 0.995, and *n*-day risk of use of ventilation with an ROC-AUC between 0.982 and 0.997. The corresponding *n*-day forecasting models predicted the needed ICU capacity with a coefficient of determination (R^2^) between 0.334 and 0.989 and use of ventilation with an R^2^ between 0.446 and 0.973. The forecasting models performed worst, when forecasting many days into the future (for large *n*). For *n* = 5, ICU capacity was predicted with ROC-AUC 0.990 and R^2^ 0.928, and use of ventilator was predicted with ROC-AUC 0.994 and R^2^ 0.854. Random Forest-based modelling can be used for accurate *n*-day forecasting predictions of ICU resource requirements, when *n* is not too large.

## Introduction

Since the outbreak of the COVID-19 pandemic in early 2020, almost 205 million confirmed cases and 4.3 million deaths have occurred as a result of the SARS-CoV-2 infection worldwide^1,2^. The speed of viral spread combined with hospital and governmental systems being ill-prepared for large-scale pandemic responses, created a situation where allocation of health care resources, including the mobilization of ad-hoc intensive care and isolation units were urgently needed.

As hospital resources were redirected towards the COVID-19 response, elective visits and planned surgical procedures quickly became the victim of collateral damage induced by the shift in health care resources, resulting in a massive backlog of elective procedures that will likely take months or even years to overcome^3^.

As expected, the pandemic hit societies in waves, creating an ebb during the summertime where resources could again be partly redirected towards other tasks. The choice of when to up or downscale the COVID-19 response thus quickly became a challenge for hospital planners, mobilizing resources at the time of pandemic acceleration while re-routing physical as well as staff-resources during the months characterized by low infection rates.

Deciding when to increase the resources for the pandemic response at the cost of the elective workload is, however, a challenge. Such decisions are dependent on accurate prediction models capable of risk-stratifying patients based on available health information on confirmation of SARS-CoV-2 infection, as well as ensuring that these predictive models can adjust to changes in disease patterns as the pandemic progresses from the first to subsequent waves of infection^4,5^. And indeed, according to a recent review by Becker et al. as of Sept 27, 2020, there have been more than 5000 modelling analyses published in peer-reviewed journals, excluding preprint servers, since the start of the epidemic^6^. In contrast to mechanistic models, we and others have trained machine learning (ML) models on electronic health record (EHR) data towards risk prediction on SARS-CoV-2 infection, specifically targeting resource-requiring events such as hospitalization, ICU admission and use of mechanical ventilation^7–9^. We found that accurate risk prediction of the individual patient (micro-prediction) can be performed with a limited EHR dataset, thus opening the potential for predicting hospital resource requirements on a population-wide scale (macro-prediction) in advance, based on available EHR data at the time of COVID-19 diagnosis.

Usual epidemiological modelling takes into account the number of infected patients, and, based on a small number of constants, partial differential equations model the future development of the epidemic ^10^. While providing insights into the role of macroscopic variables on the disease dynamics, such approaches have limited ability to model shifts in epidemic constants arising from the underlying demography of the patients as well as changes in the testing strategy. An approach based on the full medical history of all individuals tested positive for SARS-CoV-2 has the potential to overcome these issues as shifts in the epidemic constants will be reflected in the inputs, and by considering the more detailed information provided at the level of individuals—in contrast to macroscopic variables at population level—it may allow for higher accuracy. We hypothesize that such an approach modelling the epidemic severity development based on relevant clinical information can yield more precise forecasting essential for the resource management in hospitals.

Thus, this study investigates whether the previously trained ML models^9^ can be retrained for the purpose of *n*-day forecasting, capable of predicting the number COVID-19 related ICU admissions and use of mechanical ventilation in a bi-regional cohort in Denmark. While the approach is also applicable to hospital admission in theory, patients are often not known to the system in the *n-*day timeframe prior to admission (due to not yet having been tested for SARS-CoV-2), and thus the method will likely be less precise. Similarly, the approach is also applicable to the prediction of mortality within the *n*-day period, although forecasting the number of deaths explicitly is less relevant in terms of determining the need for hospital resources. However, for completeness, we investigated the approach for forecasting both hospital admission and mortality and included results in the supplementary material.

We apply our suggested approach for various values of *n* = 1, 2, …, 15, and provide a more in-depth analysis and evaluation of the 5- and 10-day forecasts, as we expect these to meet the tradeoff between a relevant window for hospital planners with perceived retention of predictive accuracy.

## Data

Data access and handling was carried out in accordance with national guidelines and regulations. The study was approved by the relevant legal boards: the Danish Patient Safety Authority (Styrelsen for Patientsikkerhed, approval #31-1521-257) and the Danish Data Protection Agency (Datatilsynet, approval #P-2020-320). Under Danish law, these agencies provide the required legal approval for the handling of sensitive patient data, including EHR data, without patient consent.

### Cohort information

We conducted the study on health data extracted from the Danish Capital and Zealand region EHR system, covering approx. 2.5 million citizens in eastern Denmark. The extracted cohort consisted of 42,526 patients tested positive for SARS-CoV-2 in both regions from March 3rd, 2020 to May 26th, 2021. Extracted patient data included age, Body Mass Index (BMI), sex, smoker/non-smoker status and diagnoses. Extracted temporal data included time and date of SARS-Cov-2 PCR tests, hospital and ICU admissions (due to COVID-19), use of mechanical ventilation, medications administered, laboratory tests performed, vital signs recorded, and death where applicable.

### Data set construction

For each patient, a timeline was constructed from the time of the first positive SARS-CoV-2 test and until censoring. Patients were censored at the time of death, a negative SARS-CoV-2 PCR test, 30 days without hospitalization (but 30 days after the positive test at the earliest), or at the time of cohort extraction.

From each patient timeline, a set of *snapshots* was constructed, one for every 24 h. Each patient snapshot consisted of the set of features extracted for the patient: age, sex, BMI, comorbidities (based on the ICD-10 and ATC codes, for a full definition, please see^9^, smoker/non-smoker status, lab tests, and temporal features: time since positive test result, time spent in hospital, time spent in ICU, and time on a ventilator. See Supplementary Table 1 for a complete overview of features included. For each lab test and vital sign feature, we added the following aggregated features to each snapshot, based on all tests/measurements prior to the timestamp of the snapshot:

- Most recent test/measurement,
- Mean of tests/measurements,
- Fitted slope of tests/measurements,
- Number of tests/measurements.

In total, 1,246,019 snapshots were created covering the set of patients in the timespan March 3rd, 2020 to May 26th, 2021.

For each patient snapshot, we defined *n*-day forecast prediction targets for ICU admission and use of mechanical ventilation. Each target described the *n*-day forecast for the patient, for instance whether the patient will be in hospital *n* days later.

By summing targets for snapshots with the same date, we obtained corresponding population wide *n*-day forecast *training targets*: The number of patients admitted to ICU *n* days from a given date, and the number of patients on mechanical ventilation *n* days from a given date.

A limitation of constructing population wide *n*-day targets in this fashion is that they underestimate the ground truth due to late arriving patients; any *n*-day target cannot include patients added to the dataset in the *n* days after the prediction is made. This problem increases with *n*. To get a proper evaluation of our approach, we thus also consider the *true targets* for each training target above, by summing over the actual admitted and ventilated patients for each date.

## Methods

### Evaluation setup

We applied *Random Forest (RF)*^11^ models for *n*-day forecasting of ICU admission and ventilator use for all patients with a positive SARS-CoV-2 test. We trained and evaluated the models in two different setups:

- Monthly retraining: To emulate the real-world use case in which models will be frequently retrained (possibly daily), we fitted models on all (training) data until the first of a month and evaluated them on data for the following month (test data), for each month starting with June 2020 and ending with May 2021. For instance, models trained on data until June 1st were evaluated on data from June. Predictions from each month were then pooled together to be used for evaluation over the entire period from June 1st until May 26th.
- First wave: The dataset was split in two: the first wave and subsequent waves (before and after June 1st). Models were fitted on the first wave (training data) and evaluated on the remaining data (test data). Predictions from the entire second data set was used for evaluation in this setting.

Note, that in both setups, some patients may be present in both training and test partitions of data. This was chosen as it reflects the real-world usage with frequent retraining, and thereby best estimates the real-world performance.

For model fitting, missing BMI measurements were imputed based on the training data using *k*-nearest neighbor imputation considering only patients of the same sex (*k* = 100). Missing in-hospital tests and measurements were set to 0.

### Model setup

We fitted random forests on the training set^11,12^, with each individual decision tree in the ensemble trained on a bootstrap sample from the training set, leaving a set of *out-of-bag (OOB) samples* available for evaluating the performance of the RF^11^.

We used 500 trees and considered all features in each split, using the Gini-impurity as splitting criterion. We tuned the *maximum tree depth* (2, 4, 6 and 8) by performing grid search using the *Receiver Operating Characteristics Area Under the Curve (ROC-AUC)* computed on the OOB samples as evaluation metric.

Population wide (macro) forecasts were obtained from the individual (micro) prediction models by summation over the predicted probabilities for all individual patients for a given timestamp. Furthermore, we also computed the 95% confidence intervals (CI) by considering individual predictions as dichotomous Bernoulli variables and then summing their variances.

### Evaluation metrics

We evaluated the population wide forecast models by computing the *coefficient of determination (R*^*2*^*)* and the *maximum error (ME)* between the predictions and the true targets. Furthermore, we constructed plots of the true target and the forecast trajectory for both ICU admissions and ventilator use, including in the plots the training targets and the 95% CIs for the outputs.

We also evaluated the micro models for making individual predictions by computing the ROC-AUC scores over all test set predictions, as well as plotting the ROC curves.

We computed 95% confidence intervals for both R^2^ and ROC-AUC scores by bootstrapping. Furthermore, the performance of the RF for predicting individual outcomes was compared to the baseline using the deLong test^13^.

### Baseline comparison models

We considered three baseline models:

- A standard *logistic regression (LogR)* micro-level model fitted to the individual patients and applied in the same fashion as the RF to make population wide forecasts. Continuous features were Z-normalized before the model was applied.
- A *static* micro-level model, which for each individual patient simply outputs the current state of the patient, that is, it predicts hospitalization if the patient is currently in hospital.
- A standard *linear regression (LinR)* macro-level model fitted directly on the true target curves (ICU admission or ventilated patients); for predicting the number of patients admitted to ICU or ventilated at day *D* + *n*, the model input was the number of patients at days *D, D-1, D-2, D-4, D-8,* and *D-12.* In contrast to our approach and the above baselines, this model was fitted on the true targets, which is possible because it works directly on the population level.

## Results

### Demographics

**Figure 1:**
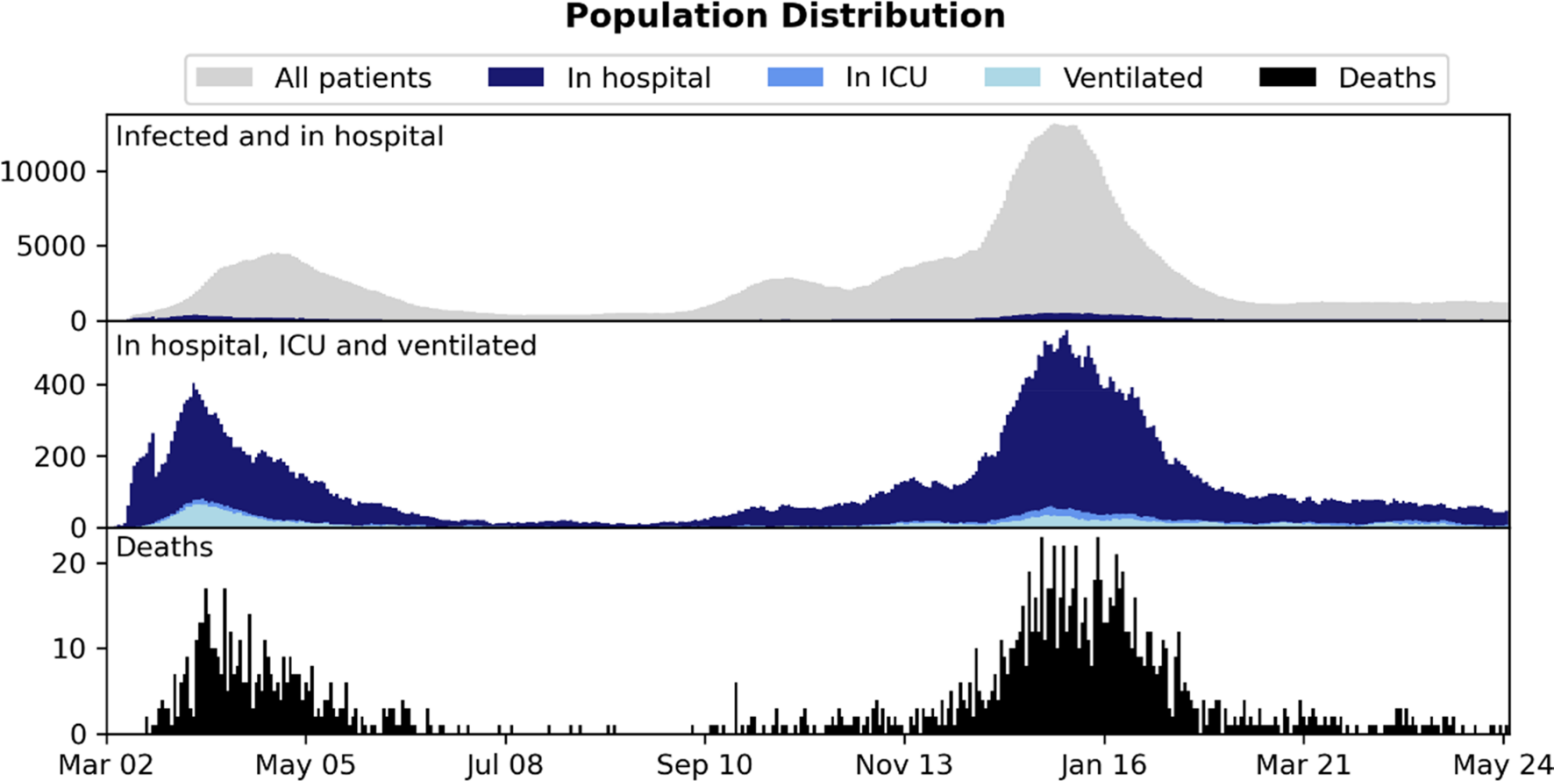
Distribution of patient 24-h snapshots during the period (March 2020–May 2021) considered in the study.

Table 1 presents an overview of the data set, including the number of hospitalized, ICU admitted and ventilated patients. Figure 1 shows the distribution of snapshots over time. Patients were observed to change state multiple times during the observed time period for both ICU and ventilator use. Table 2 reports median, interquartile range (for continuous features) and percentage (for binary features) for age, sex, BMI, and comorbidities in each of the three data sets. The percentages of missing features are listed in Supplementary Table 1, including the percentages of missing features for the subset of patients in hospital.

**Table 1:** An overview of the data sets and the number of patients in different states: patients tested positive with SARS-CoV-2, patients admitted to hospital, and patients admitted to ICU.

Patient state	#Patients	#Snapshots
Infected	42,526	1,246,019
In hospital	7271	216,685
In ICU	657	17,370

**Table 2:** Data set statistics: basic patient information (BMI, age, sex), patient comorbidities and smoker/non-smoker status.

	Infected	Not admitted to hospital	Admitted to hospital	Not admitted to ICU	Admitted to ICU	Survivor	Non-survivor
#Patients	42,526	35,255	7271	6614	657	40,872	1540
Age	44 (27–61)	39 (24–55)	70 (54–81)	70 (53–81)	68 (58–75)	43 (26–59)	83 (76–90)
Body mass index	24.7 (20.9–28.9)	24.1 (20.1–28.3)	26.1 (22.8–30.2)	25.9 (22.7–30.1)	27.6 (24.1–32.0)	24.7 (20.8–29.0)	24.4 (21.4–28.1)
Male	44.1%	42.0%	54.2%	52.6%	70.3%	43.8%	52.0%
Diabetes	8.0%	3.2%	31.1%	26.3%	79.9%	6.9%	36.4%
Ischemic heart disease	1.8%	0.6%	7.6%	7.2%	11.6%	1.6%	9.0%
Heart failure	0.1%	0.0%	0.3%	0.3%	0.3%	0.1%	0.3%
Arrhythmia	2.0%	0.5%	8.9%	8.3%	14.2%	1.4%	16.1%
Stroke	3.5%	2.1%	10.4%	10.6%	7.8%	3.0%	16.7%
Asthma	6.8%	4.2%	19.5%	19.2%	22.4%	6.2%	22.5%
Arthritis	0.7%	0.5%	1.6%	1.6%	1.7%	0.6%	2.0%
Osteoporosis	2.7%	1.6%	8.0%	8.2%	6.1%	2.4%	12.1%
Dementia	2.2%	1.7%	5.1%	5.5%	0.8%	1.8%	14.2%
Severe mental disorder	1.0%	0.8%	2.1%	2.0%	2.6%	1.0%	1.8%
Immuno-deficiencies	0.1%	0.1%	0.1%	0.1%	0.6%	0.1%	0.1%
Neurological manifestations	8.8%	7.3%	16.3%	16.5%	14.2%	8.2%	23.8%
Cancer	5.0%	3.1%	14.0%	14.2%	12.8%	4.3%	21.2%
Chronic kidney failure	1.1%	0.4%	4.8%	4.7%	5.3%	0.9%	7.3%
Dialysis	0.2%	0.1%	1.0%	1.0%	1.2%	0.2%	1.1%
Hypertension	13.6%	6.4%	48.7%	45.9%	77.0%	12.0%	56.5%
Smoker	17.7%	18.2%	16.7%	17.1%	12.5%	17.8%	17.6%

For binary variables, we report the occurrence in percent; for continuous variables, we report the median and interquartile range.

### Monthly retrained

**Figure 2:**
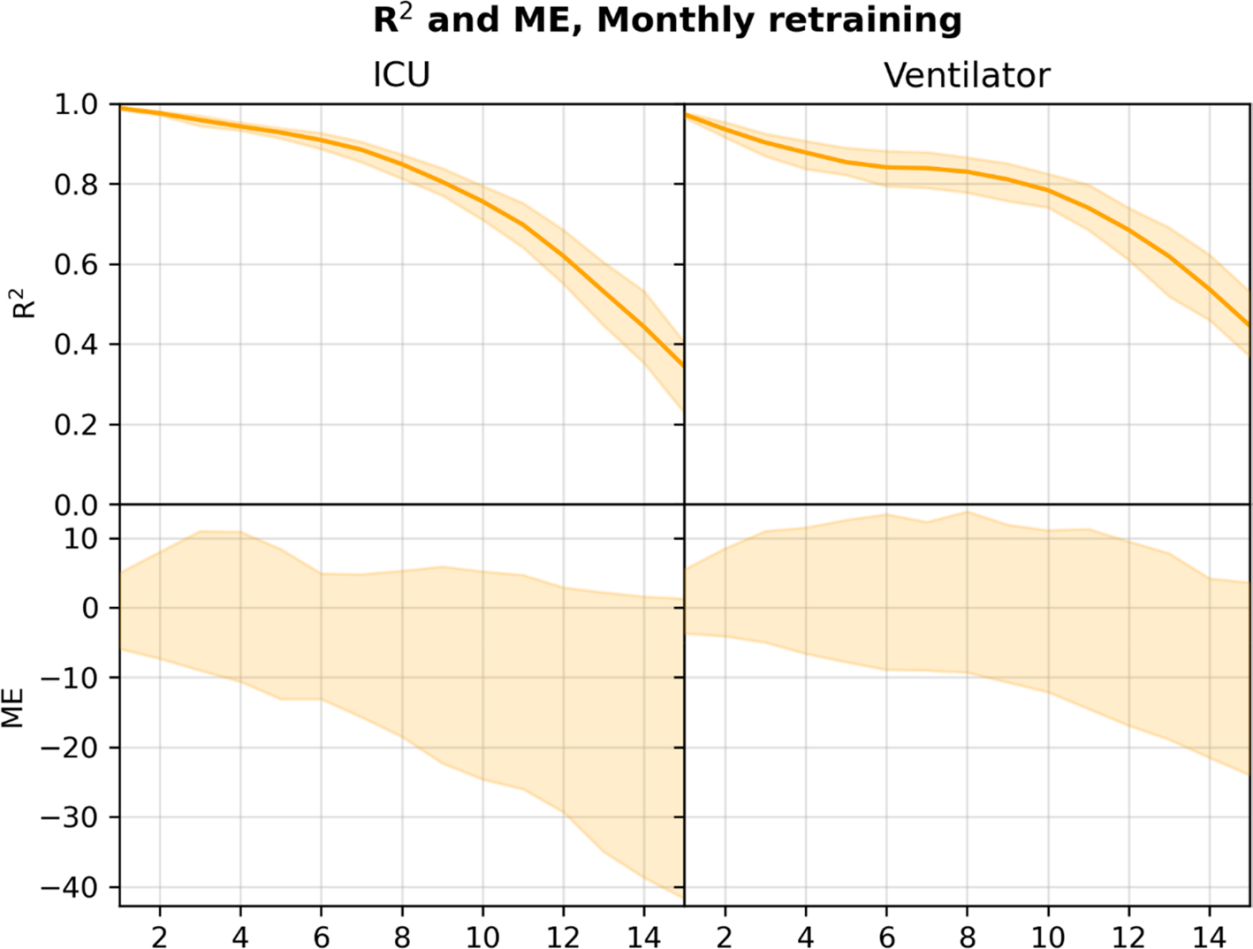
R^2^ and 95% confidence interval (top), and ME (bottom) for *n*-day forecasts using the RF model in the monthly retrained setting, computed on the pooled predictions for the months after June 2020. Numerical results are given in Supplementary Tables 3 and 4.

**Figure 3:**
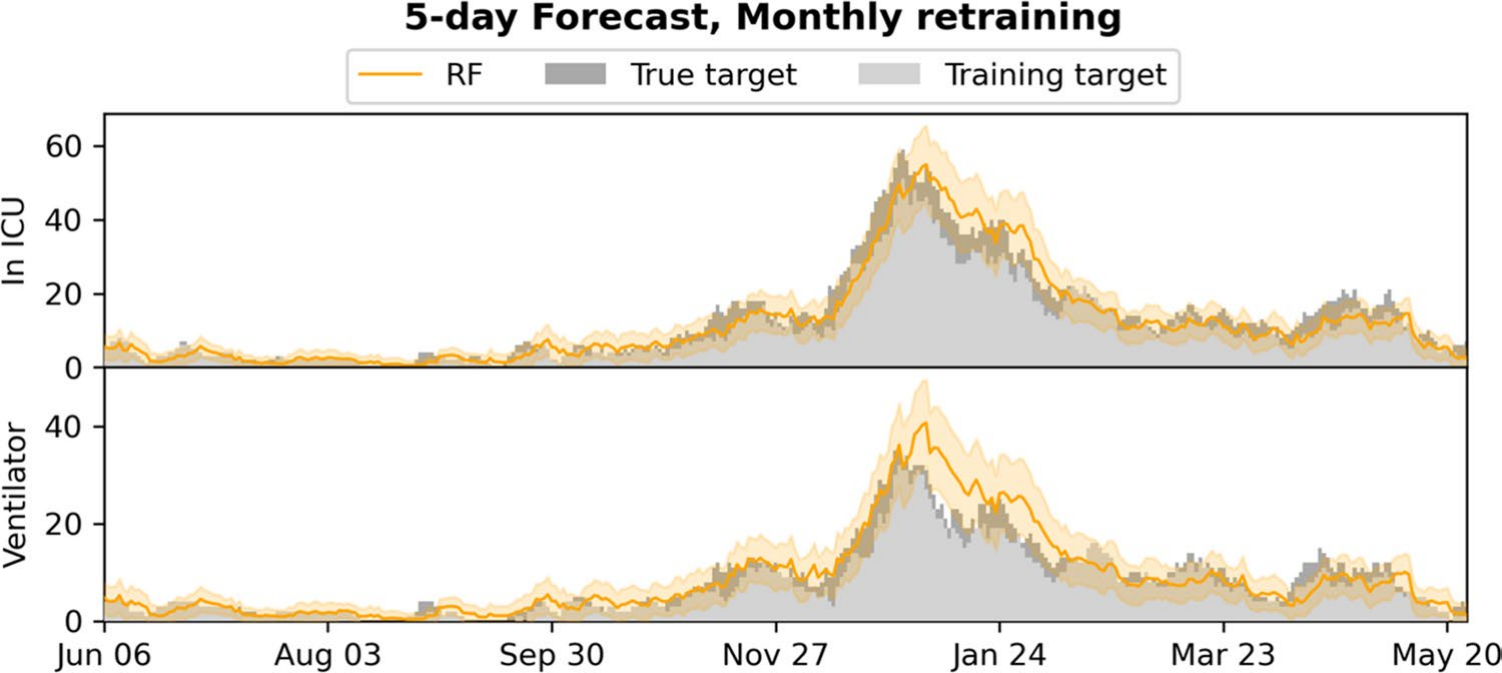
5-day forecasts of admission to ICU and use of mechanical ventilation, compared to the training and true targets in the monthly retrained setting. The estimated 95% confidence intervals are shown.

**Figure 4:**
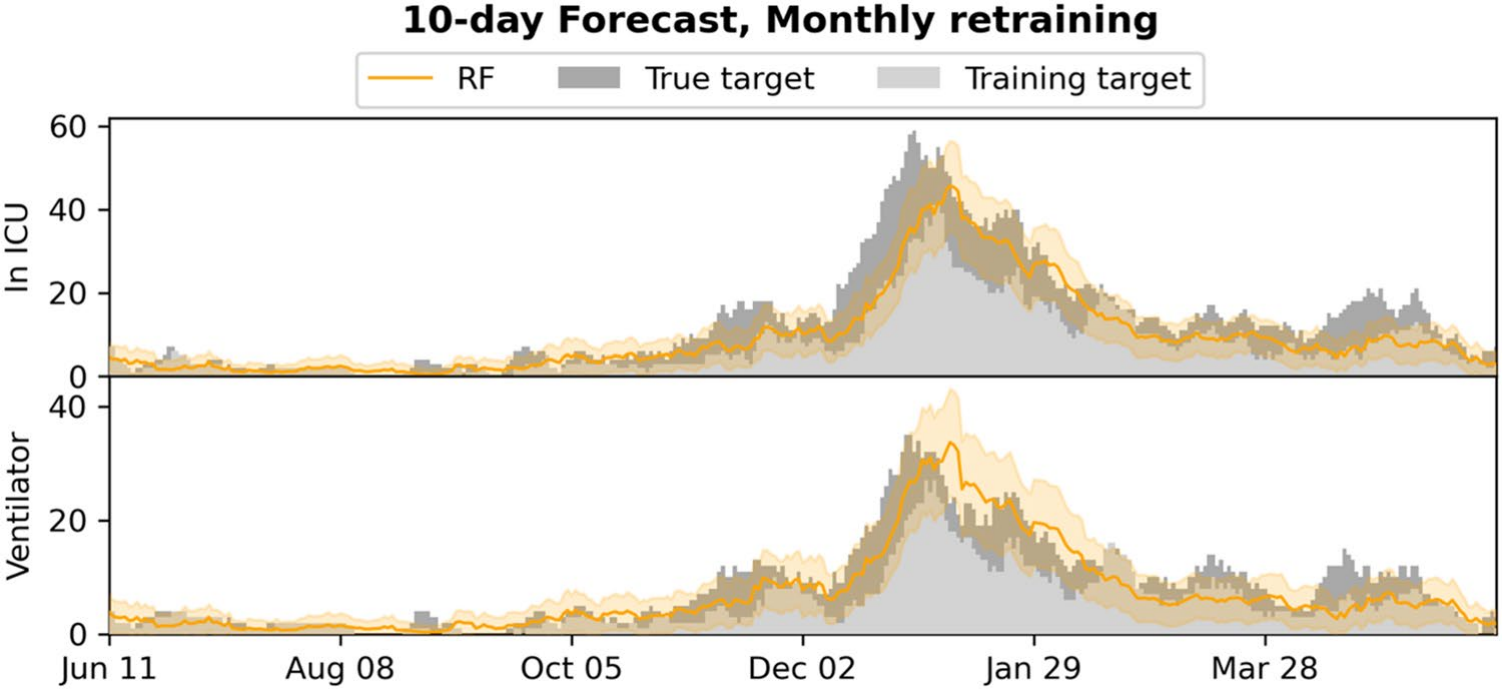
10-day forecasts of admission to ICU and use of mechanical ventilation, compared to the training and true targets in the monthly retrained setting. The estimated 95% confidence intervals are shown.

Figure 2 plots the R2 and ME for the *n-*day forecasts (*n* = 1, 2, …, 15) for the RF model. Figures 3 and 4 show the 5- and 10-day forecasts versus the training and the true targets respectively. The RF was found to perform very well when retrained monthly when *n* is not too large. As *n* increased, the performance decreased. In particular, the RF model obtained an R^2^ of 0.928 and 0.854 for 5-day forecasting of ICU admission and ventilator use respectively, with performance dropping to 0.756 and 0.784 for 10-day forecasts.

Supplementary Figs. 1 and 2 plot the baseline forecasts for *n-*day forecasts respectively (*n* = 1, 2, …, 15). Supplementary Tables 2, 3, 4, and 5 present numerical results for the RF model and the baselines, including also results for hospital admission (Supplementary Table 2) and mortality (Supplementary Table 5), with targets constructed in a similar fashion to the targets for ICU and ventilator. In general, RF performed better than the baseline models for predicting ICU admission and ventilation, when *n* was small. In particular, RF outperformed all other models on both targets for 5-day forecasts. For 10-day ICU admission forecasts, the RF exhibited similar performance to the LinR model and was outperformed by the LogR model.

### First wave

**Figure 5:**
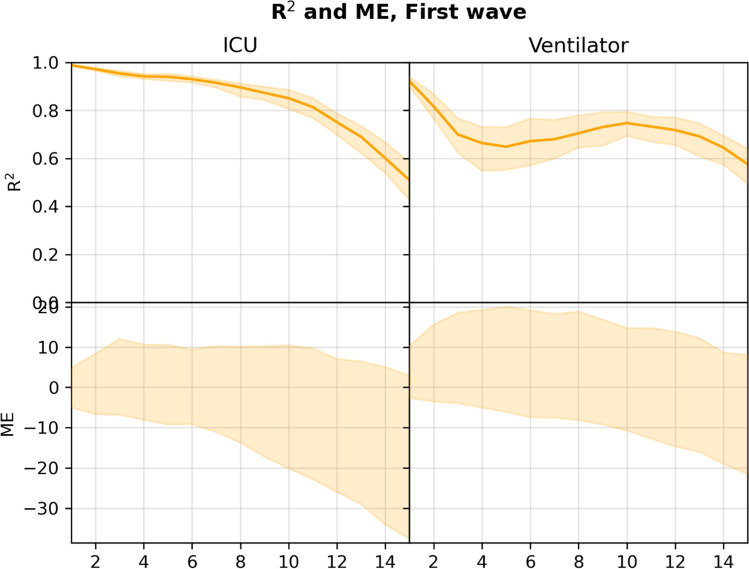
R^2^ and 95% confidence interval (top), and ME (bottom) for *n*-day forecasts using the RF model in the first wave setting, computed on the pooled predictions for the months after June 2020. Numerical results are given in Supplementary Tables 7 and 8.

**Figure 6:**
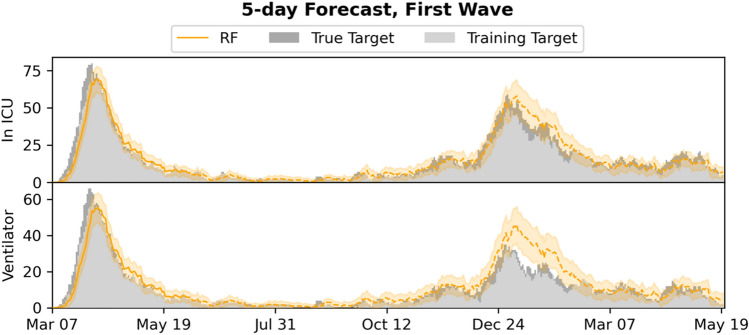
5-day forecasts of admission to ICU and use of mechanical ventilation, compared to the training and true targets in the first wave setting. Predictions and targets for both the first wave (training data) and the subsequent waves (test data, dashed) are shown, as well as the 95% confidence intervals.

**Figure 7:**
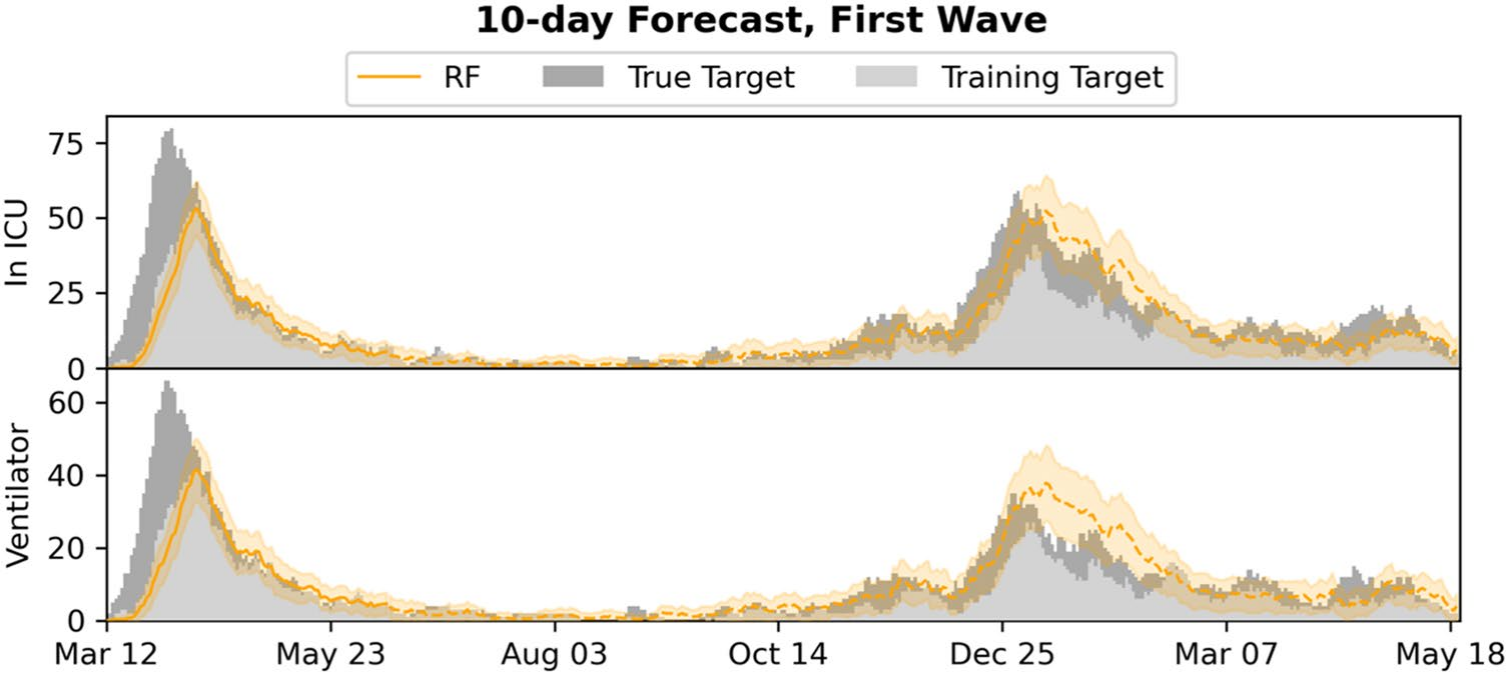
10-day forecasts of admission to ICU and use of mechanical ventilation, compared to the training and true targets in the first wave setting. Predictions and targets for both the first wave (training data) and the subsequent waves (test data, dashed) are shown, as well as the 95% confidence intervals.

Figure 5 plots the R^2^ and ME for the *n*-day forecasts (*n* = 1, 2, …, 15) for the RF model. Figures 6 and 7 show the 5- and 10-day forecasts versus the training and true targets for both the first wave (training data) and the subsequent waves (test data). The model was found to generalize well to the subsequent waves, with performance in general decreasing as *n* was increased. For 5- and 10-day ICU admission forecasts, the model obtained R^2^s of 0.940 and 0.851 respectively. For ventilator use, the corresponding R^2^s were 0.649 and 0.747, showing an increase with *n*.

Supplementary Figs. 3 and 4 plot the baseline forecasts for the 5- and 10-day forecasts respectively. Supplementary Tables 6, 7, 8, and 9 present numerical results for the RF model and the baselines, including results for hospital admission (Supplementary Table 6) and mortality (Supplementary Table 9). The RF outperformed all baselines for predicting ICU admission for *n* < 12, while the static model was in general better for predicting ventilator use for small *n*. For 5- and 10-day ICU admission, RF performed best (without overlap of the 95% CIs); in particular it outperformed the population level LinR model.

### Individual forecasts

Supplementary Table 10 reports ROC-AUC for the individual *n*-day predictions (*n* = 1, 2, …, 15) for the macro models RF and LogR when retrained monthly, including results for prediction of hospital admission and mortality. Supplementary Figs. 5 and 6 show the ROC curves for *n* = 5, 10. For all *n*-day forecasting targets, the RF was found to obtain ROC-AUC scores above 0.95 and was found to perform significantly better than LogR (*p* < 0.0001) by application of the deLong test.

## Discussion

In this study, we demonstrate how COVID-19 micro-predictions made at the level of the individual SARS-CoV-2 positive patient, can be extrapolated to a population-wide macro-prediction by modelling incremental patient data as this becomes available in the EHR system. Based on evaluation in a cohort with 42,526 SARS-CoV-2 positive patients, we find that, using an RF, accurate *n*-day forecasting of ICU admissions and ventilator use can be achieved using initial datapoints including age, sex, BMI and comorbidities with additional datapoints including lab tests and vital parameters added as these become available. As expected, the results generally degraded as *n* was increased, but models provided good fits for *n* = 5 and even *n* = 10. When using regular retraining, the RF approach generally outperformed all baseline methods; among 5- and 10-day forecasts, only logistic regression performed better for 5-day ICU admission forecasts, as explained below. The setting with regular retraining best resembles the expected real-life deployment, during which the model will be retrained on the latest available data even more frequently. When only a single model trained on the first wave was used, the RF still generalized well for *n*-day forecasting admission to ICU and use of ventilation, beating most baselines when *n* < 12, including the macro-level LinR model, confirming that, in this case, the micro-to-macro approach handles shifts in the distribution (on the macro/population level) better.

While we reported results for only monthly retraining, the data processing and RF implementation is so efficient that the models can be retrained on the entire cohort daily as new data arrives, allowing for real-time deployment of the most up-to-date models.

The system is currently limited by missing access to early SARS-CoV-2 tests, which means patients are not included in the input early, leading to the model producing underestimates of the true admission/ventilation numbers. This can be seen by inspection of the plots of the forecast trajectories and training/true targets (e.g., Figs. 2 and 3). The model consistently underestimates the true targets due to the training targets being underestimates themselves. The problem increases with the number of days *n* we look ahead in the forecasts, as a patient is less likely to be in the system, when predicting many days ahead; this can also be seen in Figs. 2 and 5, where the R^2^ score degrades. We also evaluated the model for prediction of hospital admissions, but since many patients are not confirmed positive until after admission, the issue was even more pronounced, and the model severely underestimated the true admission numbers (see Supplementary Tables 2 and 6), even though the model performed well for predicting for individual patients.

However, our focus is on ICU admission and use of ventilation, because constraints on ICU and ventilators are one of the main issues in capacity planning in hospitals^14^– and for *n* sufficiently small, for instance for the 5- and 10-day ICU and ventilator forecasts, the training targets and the true targets were fairly close, leading to good model performance and useable forecasts, with true targets often within the 95% CI.

When looking at the prediction accuracy on the individual level, LogR performed worse than RF. In our experiments, the logistic regression had a tendency to overestimate the individual risks, that is, the training targets. The training targets themselves being underestimates of the true targets, this sometimes led to LogR giving better results on the true values, despite being less accurate on the individual level; for instance, for 10-day forecasting of ICU admission.

The problem that the training targets underestimate the true targets may be reduced by access to earlier SARS-CoV-2 testing. While the Danish test capacity during and after the second wave was improved and patients in general have been tested early (before hospital admission), we only have access to tests performed by the regional test system and not by private vendors or the national test centers. Thus, we only have access to a limited number of positive tests before admission and for many of the patients we only have a confirmatory in-hospital test, leading to a very short time between test and hospital admission, and thus the discrepancy between the targets and actual number of ICU admitted or ventilated patients.

We expect the accuracy of the micro-to-macro approach to increase with access to these early tests, which might even make hospital admission forecasting feasible, however this will rarely be possible due to the number of different providers of COVID-19 tests. A more feasible alternative would be adjustment of predictions by use of macro model predictions, for instance by estimation of the total number of infected expected to arrive within the *n*-day forecasting window.

The findings mirror previously published results from the United Kingdom COVID-19 Capacity Planning and Analysis System (CPAS)^15^. In contrast to the CPAS system, our model captures patients at the time of their positive SARS-CoV-2 test result, with the ability to extract EHR information for all patients irrespective of hospital admission status. Even when we lack access to the early tests for many patients, this allows us to forecast ICU admission and ventilator use, even for non-hospitalized patients. This approach is feasible due to data sharing between the EHR system and Danish national registries, where detailed information on diagnoses codes, previous hospital admissions and pharmaceutical use is available for all Danish citizens. As such, the system can model basic demographic and healthcare datapoints even for non-hospitalized patients, which aids the prediction of need for hospital admission on a population-wide scale. However, as mentioned, the approach is limited by patients with late positive test results. In practice, the hospitalization model has to be adjusted, for instance by extending the model by modelling COVID-19 spread as done by CPAS and then adjusting the hospitalization model accordingly. Even better would be access to the early Danish PCR tests performed outside of the hospitals, which we hypothesize will alleviate the problem.

Our approach differs from conventional epidemiological analyses, where modelling is based on the number of patients tested positive, calculated infection rates and social mobility models for a specific infection during a pandemic with subsequent forecasting^16^. The generalization ability and performance of the RF model between the first and subsequent COVID-19 waves of 2020 and 2021 show the potential of modelling risk predictions at the individual level based on EHR data with subsequent extrapolation to population wide modelling in this as well as future pandemics. Furthermore, the approach could be ported to other disease settings, including a hospital-wide ICU admission forecasting, provided relevant disease modalities could be modelled by dedicated ML systems.

Pivotal for the success of this approach is, however, an ability to rapidly mobilize EHR data for the purpose of ML modelling, thus indicating the need for maintaining updated data transfer protocols from the EHR system to a High-Performance Computing (HPC) platform capable of training ML models in a secure environment. Such EHR and HPC integration should ideally be maintained even between pandemic surges, owing to the often-rapid spread and unknown features of pandemics.

Furthermore, accumulating data indicates that the patient characteristics of the COVID-19 pandemic change from the first to subsequent waves. Indeed, reports have indicated that the second wave was characterized by an altered disease spectrum and updated treatment protocols^17^, resulting in potentially milder disease trajectories with lower ICU and ventilator requirements^5^, while the current third wave is expected to be affected by the increasing rate of vaccination in the Danish population. As such, effective forecasting models need to be robust towards demographic fluctuations, which is exemplified in our approach, where the predictive performances of the models were retained for both first, second and third wave patients in this study. Furthermore, these fluctuations can be addressed by frequent model retraining, as evidenced by the improved performance of the monthly retraining method demonstrated here. While demographics may change, the pandemic progression carry the inherent risk of mutations in the viral genome altering the disease severity, as is seen SARS-CoV-2 variations such as the D614G and B117, the latter being the dominant strain in the current third wave of the pandemic in both Denmark and neighboring countries^18,19^. As such, predictive models could ideally be dynamically retrained on emerging data from multiple data sources, including potentials such as viral sequencing data in combination with EHR data.

Furthermore, adding additional data feeds (e.g., imaging data) to hybrid prediction models could potentially augment the value of such systems, which has been suggested by previous studies^20–22^. The caveat is, however, that such systems require integration of healthcare and population specific data, enabling extraction of EHR data on a community wide rather than a hospital specific scale. Healthcare systems operating as individual units rather than covering the entirety of a regional population, may thus not be optimally suited for real-time deployment of these prediction models.

This study has several limitations. First, we model data retrospectively and have not demonstrated a prospective value in this study. As such, although accurate on retrospective data predictions, novel features of a potential next COVID-19 wave could affect model performance. Furthermore, we model a selected subset of patient-derived variables based on previous experience^9^, although other data points could affect the model’s classification ability.

Secondly, we have not performed external validation on a separate cohort. This, however, may not be desirable due to several factors: The model should be trained to forecast locally, and transferring to different healthcare systems with different social and geographical factors would require retraining of the model to capture these effects. We have also recently demonstrated that transferring COVID-19 ML prediction models between health care systems internationally results in a reduction of the classification precision, presumably due to inherent differences between healthcare systems even though patient demographics are comparable^9^.

Thirdly, as mentioned, the model could benefit from either adjustment based on macro predictions, or access to earlier SARS-CoV-2 tests for Danish patients, before deployment. This might also make hospital admission feasible.

In conclusion, this study demonstrates that transferring ML based patient level predictions for COVID-19 to a population wide-scale for the purpose of *n*-day resource use predictions, is feasible and should be considered for the current and future pandemic events.

## Supplementary Information

Supplementary Information.

## Data Availability

Data used for the purpose of this manuscript cannot be publicly shared due to patient confidentiality issues.
