# Supplementary material for: Using machine learning for predicting intensive care unit resource use during the COVID-19 pandemic in Denmark
Source: Sci Rep. 2021 Sep 23;11:18959. doi: 10.1038/s41598-021-98617-1 (PMC8460747; doi:10.1038/s41598-021-98617-1)
Supplement: Supplementary file 1 — Supplementary Information. [file 41598_2021_98617_MOESM1_ESM.docx]

**SUPPLEMENTARY FIGURES**

**Supplementary Figure 1:** Plot of the 5-day forecasts of admission to ICU and use of mechanical ventilation for the different baselines in the monthly retrained setting. Training and true targets are shown, as well as estimated 95% confidence intervals for LogR (note that LinR and static do not admit a 95% CI).


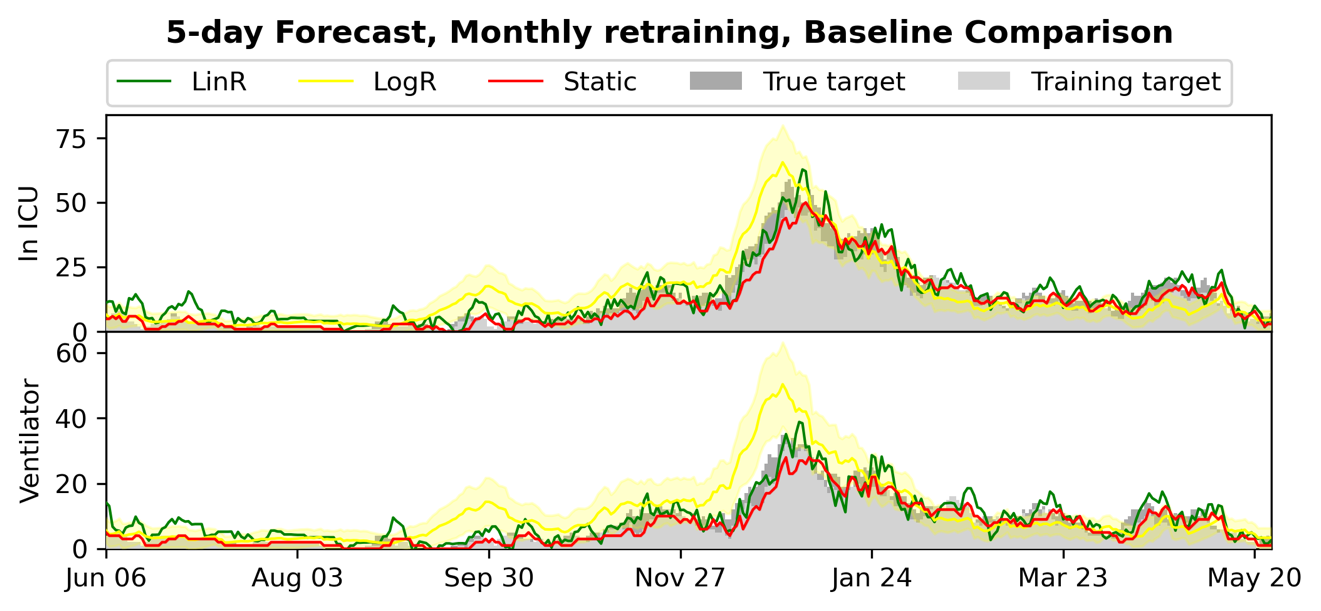


**Supplementary Figure 2:** Plot of the 10-day forecasts of admission to ICU and use of mechanical ventilation for the different baselines in the monthly retrained setting. Training and true targets are shown, as well as estimated 95% confidence intervals for LogR.


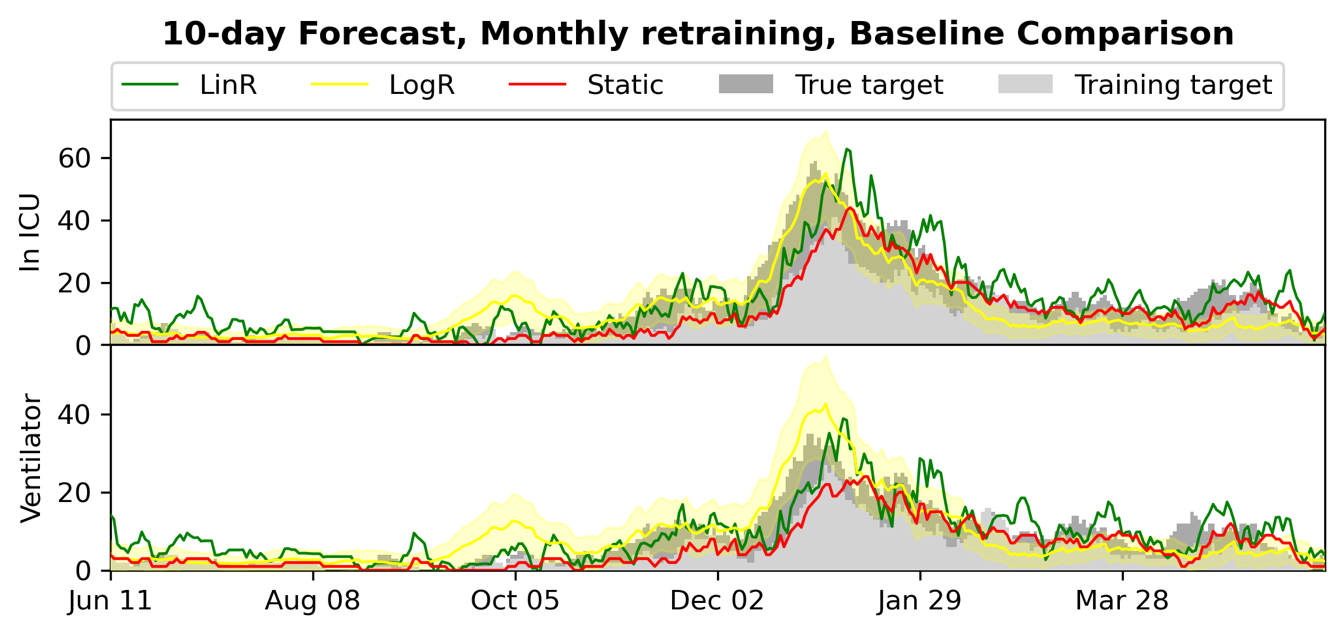


**Supplementary Figure 3:** Plot of the 5-day forecasts of admission to ICU and use of mechanical ventilation for the different baselines in the first wave setting. Predictions, training and true targets are shown for both the first wave (training data) and the second wave (test data), as well as estimated 95% confidence intervals for LogR.


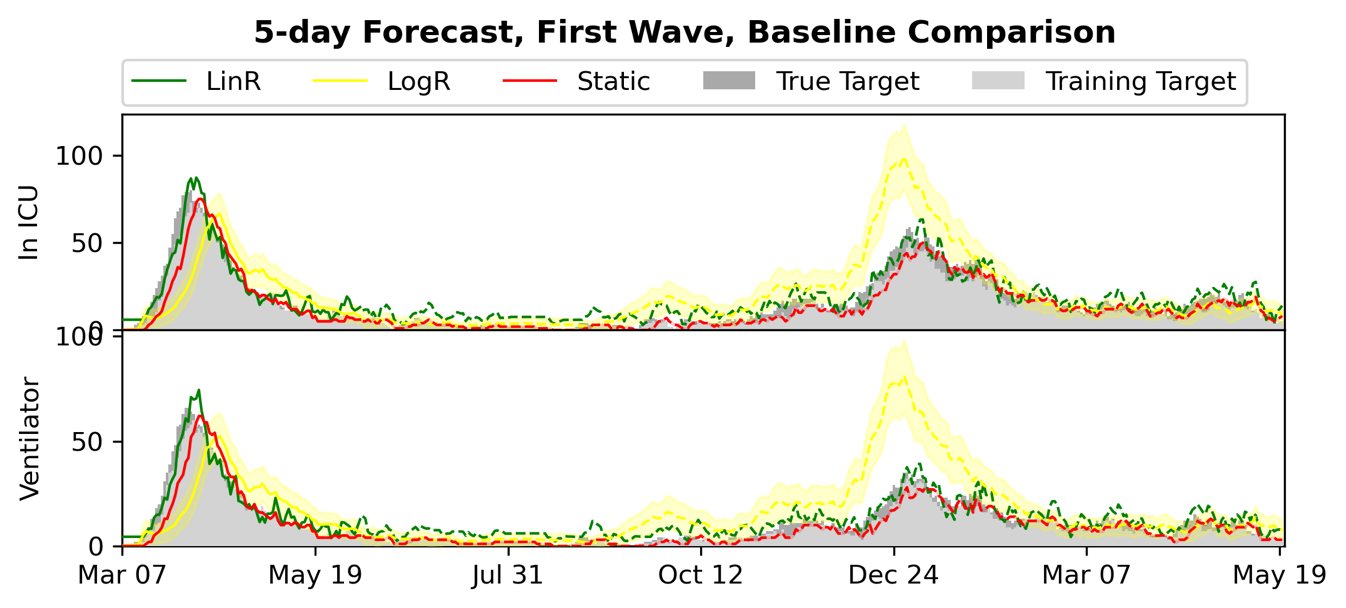


**Supplementary Figure 4:** Plot of the 10-day forecasts of admission to ICU and use of mechanical ventilation for the different baselines in the first wave setting. Predictions, training and true targets are shown for both the first wave (training data) and the second wave (test data), as well as estimated 95% confidence intervals for LogR.


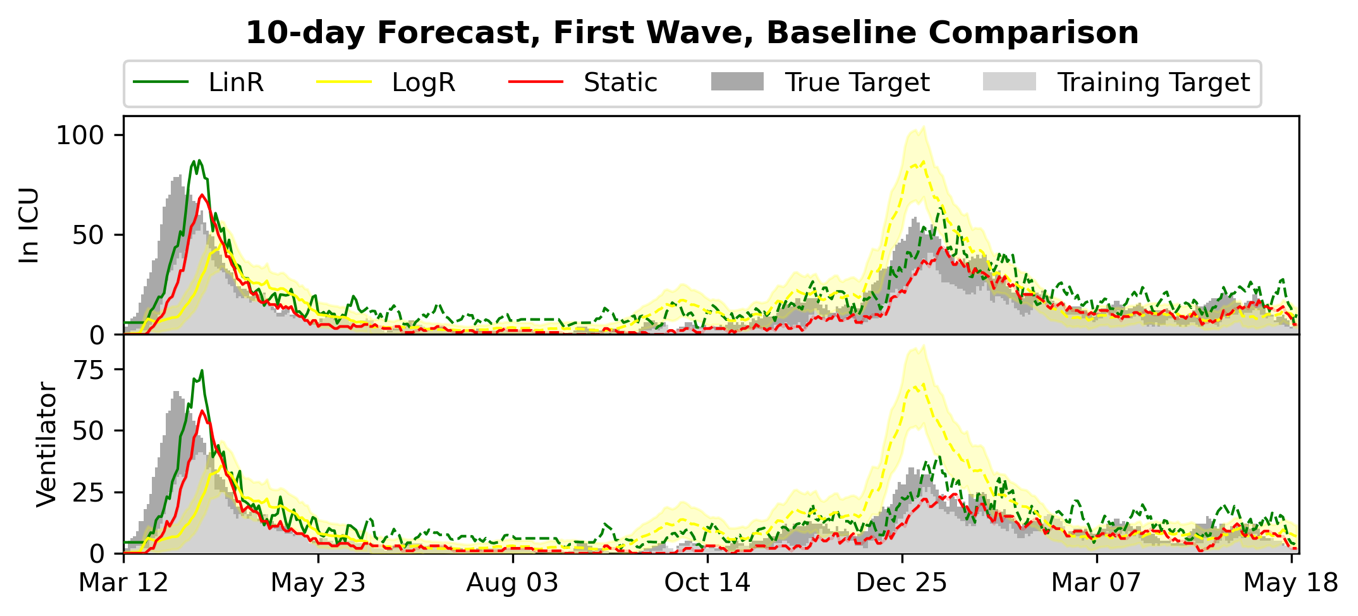


**Supplementary Figure 5:** ROC curves for evaluation of the individual 5-day RF and LogR predictions in the monthly retrained setting.


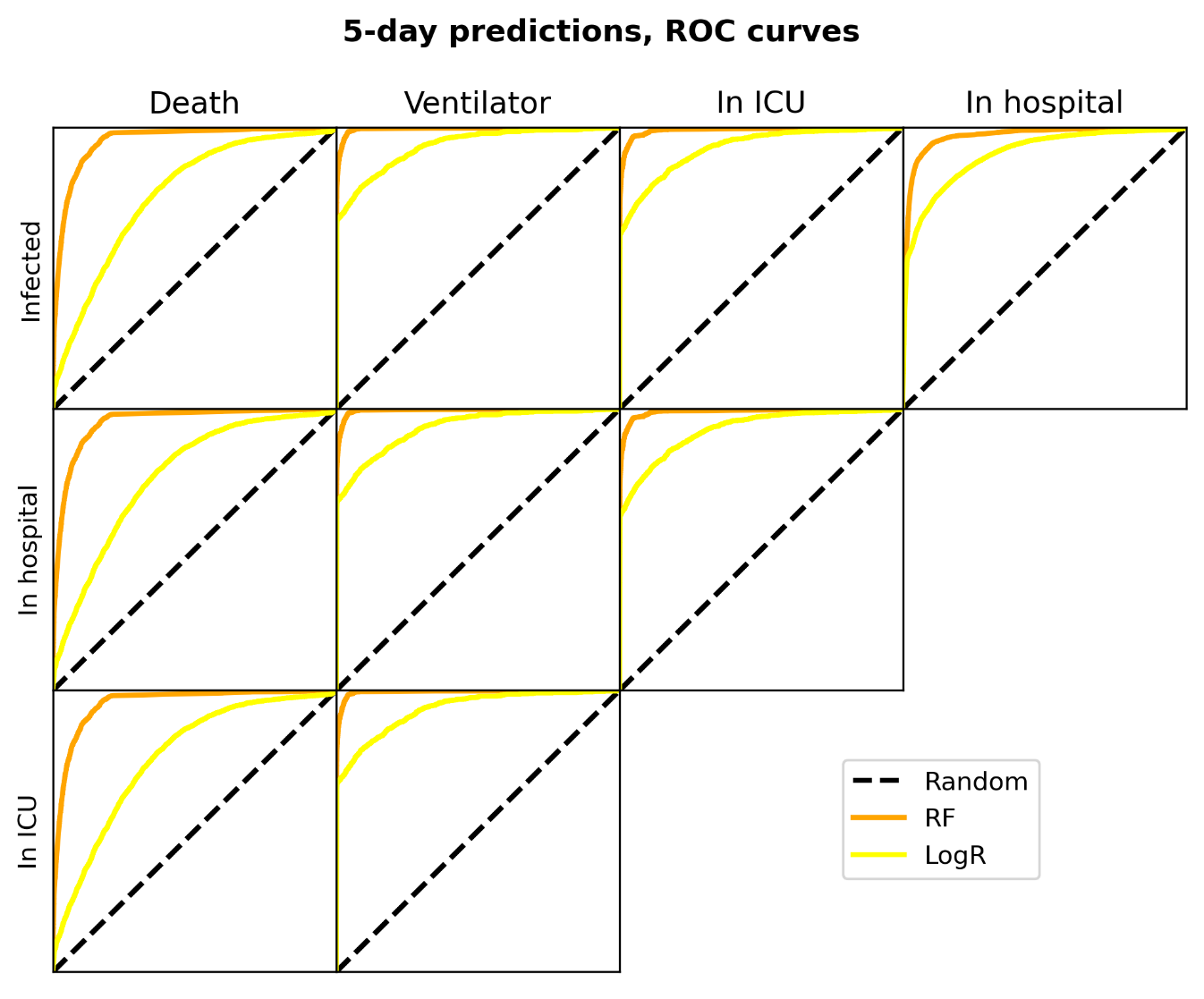


**Supplementary Figure 6:** ROC curves for evaluation of the individual 5-day RF and LogR predictions in the monthly retrained setting.


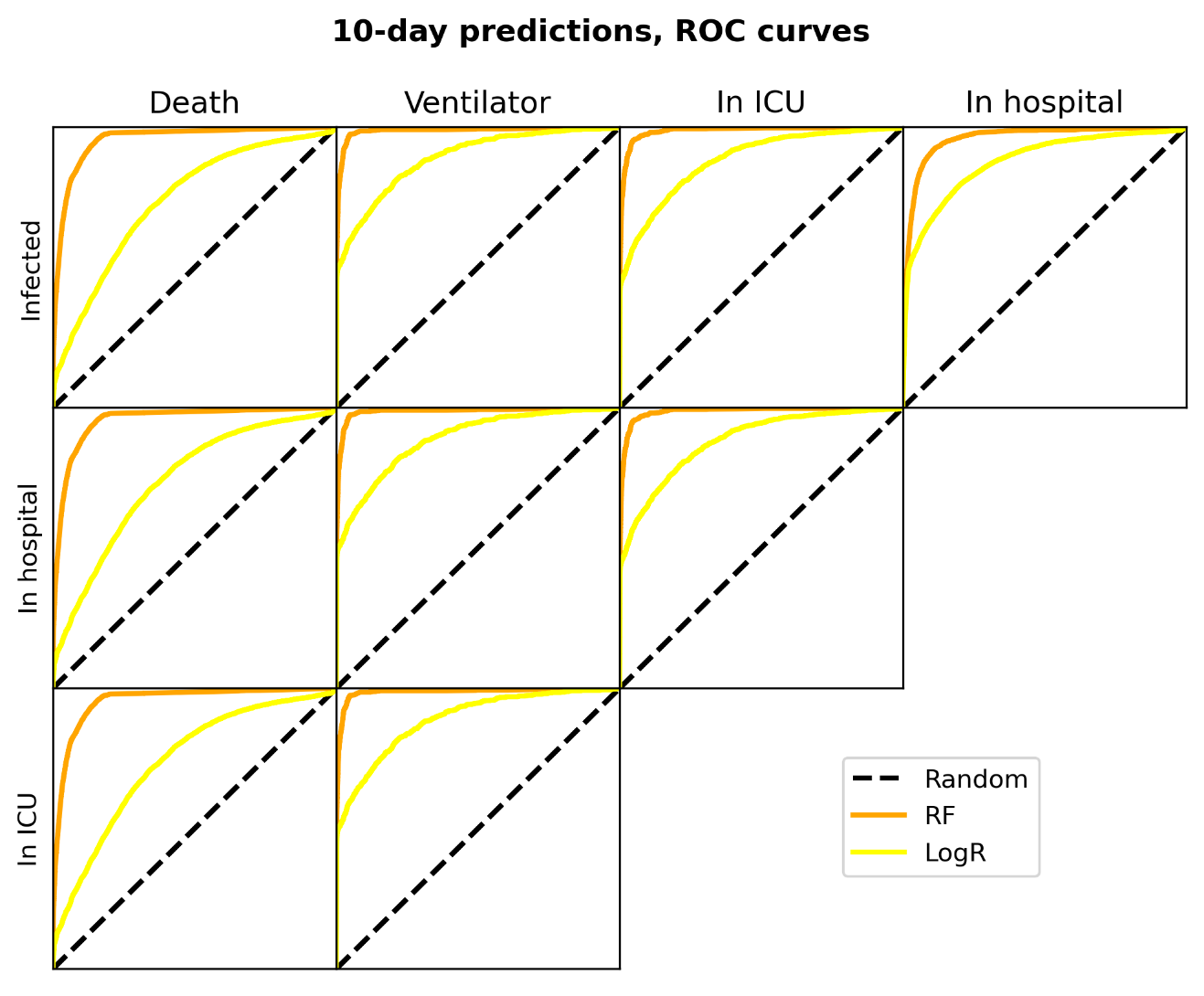


**SUPPLEMENTARY TABLES**

**Supplementary Table 1:** Feature categories, information, feature names and percentage missing features. For missing features, the percentages of missing features for all infected patients and the subset consisting of hospitalized patients are reported.

| **Category** | **Feature** | **Missing** | **Missing (hospital)** |
| --- | --- | --- | --- |
| **Basic.** Continuous (except Male Sex) | Age | 0% | 0% |
|  | Body Mass Index | 49.9% | 16.2% |
|  | Male Sex | 0% | 0% |
| **Comorbidities.** Binary. Extracted based on ICD-10 codes and medication. | Diabetes | 0% | 0% |
|  | Ischemic heart disease | 0% | 0% |
|  | Heart failure | 0% | 0% |
|  | Arrhythmia | 0% | 0% |
|  | Stroke | 0% | 0% |
|  | Asthma | 0% | 0% |
|  | Arthritis | 0% | 0% |
|  | Osteoporosis | 0% | 0% |
|  | Dementia | 0% | 0% |
|  | Severe mental disorder | 0% | 0% |
|  | Immunodeficiencies | 0% | 0% |
|  | Neurological manifestations | 0% | 0% |
|  | Cancer | 0% | 0% |
|  | Chronic kidney failure | 0% | 0% |
|  | Dialysis | 0% | 0% |
|  | Hypertension | 0% | 0% |
| **Habits.** Binary. | Is smoker | 63.2% | 35.0% |
| **Temporal.** Continuous. | Time infected | 0% | 0% |
|  | Time in hospital | 82.9% | 0% |
|  | Time in ICU | 98.5% | 91.0% |
|  | Time in ventilator | 99.0% | 94.1% |
| **Labaratory tests.** For each test, four aggregated, continuous features were computed based on all measurements before a snapshot: count, mean, slope, and most recent. | CRP | 86.5% | 29.3% |
|  | Lymphocyte Count | 87.4% | 33.4% |
|  | Lactic dehydrogenase | 88.5% | 39.2% |
|  | Alanine aminotransferase | 88.1% | 37.2% |
|  | Hemoglobin | 86.5% | 28.6% |
|  | White blood cells | 86.6% | 29.3% |
|  | Neutrophil count | 90.6% | 51.1% |
|  | D dimer | 93.3% | 62.6% |
|  | Blood urea nitrogen | 88.3% | 38.2% |
|  | Creatinine | 86.6% | 29.4% |
|  | Ferritin | 92.7% | 58.9% |
|  | Base excess | 97.5% | 85.3% |
|  | HCO₃ | 96.5% | 80.0% |
|  | Lactate | 98.3% | 90.2% |
|  | O₂ | 96.6% | 80.4% |
|  | pCO₂ | 96.6% | 80.4% |
|  | ph | 97.5% | 85.6% |
|  | pO₂ | 96.7% | 80.4% |
| **Vital signs.** For each test, four aggregated, continuous features were computed based on all measurements before a snapshot: count, mean, slope, and most recent. | Pulse | 80.3% | 8.6% |
|  | Temperature | 80.6% | 9.8% |
|  | Early Warning Score | 82.4% | 13.9% |
|  | Respiratory rate | 80.7% | 10.3% |
|  | Saturation | 80.3% | 8.7% |

**Supplementary Table 2:** *n*-day hospital admission forecasting results for the RF and baseline models in the monthly retrained setting. R^2^ and 95% CIs (top), and ME (bottom) were computed on the pooled predictions for the months after June 2020.

| **Hospital admission** | | | | |
| --- | --- | --- | --- | --- |
| *n* | RF | LogR | LinR | Static |
| 1 | 0.982 (0.979 - 0.985) | 0.932 (0.922 - 0.942) | 0.961 (0.945 - 0.969) | 0.983 (0.979 - 0.986) |
|  | -81.4, 4.3 | -153.5, 93.0 | -111.6, 70.9 | -88.0, 20.0 |
| 2 | 0.942 (0.93 - 0.95) | 0.894 (0.875 - 0.908) | 0.951 (0.939 - 0.96) | 0.97 (0.964 - 0.978) |
|  | -128.4, 4.0 | -204.3, 63.0 | -130.6, 94.9 | -98.0, 36.0 |
| 3 | 0.892 (0.875 - 0.903) | 0.85 (0.821 - 0.874) | 0.942 (0.924 - 0.954) | 0.956 (0.945 - 0.969) |
|  | -164.9, 4.3 | -222.9, 46.6 | -126.7, 105.9 | -131.0, 54.0 |
| 4 | 0.83 (0.812 - 0.845) | 0.797 (0.768 - 0.826) | 0.933 (0.921 - 0.946) | 0.941 (0.924 - 0.953) |
|  | -204.8, 2.7 | -246.9, 44.8 | -136.7, 137.3 | -140.0, 58.0 |
| 5 | 0.759 (0.722 - 0.787) | 0.732 (0.694 - 0.759) | 0.927 (0.908 - 0.941) | 0.926 (0.905 - 0.943) |
|  | -229.4, 3.8 | -264.0, 41.3 | -146.7, 146.3 | -149.0, 74.0 |
| 6 | 0.681 (0.643 - 0.707) | 0.657 (0.621 - 0.687) | 0.921 (0.893 - 0.942) | 0.91 (0.888 - 0.931) |
|  | -255.5, 3.0 | -278.9, 35.5 | -159.7, 143.3 | -164.0, 66.0 |
| 7 | 0.593 (0.561 - 0.623) | 0.57 (0.542 - 0.601) | 0.908 (0.882 - 0.927) | 0.891 (0.861 - 0.915) |
|  | -293.2, 3.0 | -293.7, 26.7 | -175.7, 123.9 | -189.0, 75.0 |
| 8 | 0.503 (0.459 - 0.532) | 0.483 (0.441 - 0.519) | 0.887 (0.86 - 0.911) | 0.866 (0.826 - 0.895) |
|  | -324.9, 3.3 | -315.4, 18.7 | -199.7, 151.9 | -211.0, 93.0 |
| 9 | 0.411 (0.35 - 0.463) | 0.396 (0.347 - 0.438) | 0.86 (0.822 - 0.893) | 0.839 (0.802 - 0.883) |
|  | -351.4, 2.0 | -330.1, 10.0 | -221.7, 174.9 | -232.0, 109.0 |
| 10 | 0.321 (0.264 - 0.371) | 0.311 (0.269 - 0.36) | 0.833 (0.792 - 0.86) | 0.81 (0.758 - 0.847) |
|  | -372.0, 1.4 | -346.6, 3.3 | -240.7, 186.9 | -248.0, 119.0 |
| 11 | 0.23 (0.162 - 0.285) | 0.227 (0.173 - 0.28) | 0.808 (0.76 - 0.849) | 0.781 (0.717 - 0.815) |
|  | -392.0, 0.9 | -362.6, 2.8 | -243.7, 188.9 | -255.0, 132.0 |
| 12 | 0.141 (0.075 - 0.192) | 0.142 (0.087 - 0.193) | 0.784 (0.727 - 0.829) | 0.751 (0.706 - 0.808) |
|  | -412.0, 0.2 | -375.2, 2.2 | -237.7, 192.9 | -276.0, 142.0 |
| 13 | 0.052 (-0.006 - 0.128) | 0.058 (0.007 - 0.108) | 0.76 (0.702 - 0.815) | 0.72 (0.651 - 0.792) |
|  | -432.4, 0.0 | -401.5, 1.6 | -280.2, 189.9 | -305.0, 139.0 |
| 14 | -0.032 (-0.108 - 0.023) | -0.022 (-0.091 - 0.036) | 0.733 (0.675 - 0.776) | 0.688 (0.622 - 0.745) |
|  | -448.3, -0.3 | -422.2, 0.6 | -304.7, 188.9 | -320.0, 120.0 |
| 15 | -0.113 (-0.187 - -0.053) | -0.096 (-0.169 - -0.034) | 0.697 (0.612 - 0.754) | 0.653 (0.559 - 0.724) |
|  | -464.7, -0.9 | -438.8, 0.1 | -318.2, 191.9 | -348.0, 127.0 |

**Supplementary Table 3:** *n*-day ICU admission forecasting results for the RF and baseline models in the monthly retrained setting. R^2^ and 95% CIs (top), and ME (bottom) were computed on the pooled predictions for the months after June 2020.

| **ICU admission** | | | | |
| --- | --- | --- | --- | --- |
| *n* | RF | LogR | LinR | Static |
| 1 | 0.989 (0.986 - 0.991) | 0.929 (0.91 - 0.942) | 0.923 (0.901 - 0.939) | 0.986 (0.982 - 0.988) |
|  | -5.9, 5.0 | -8.7, 14.8 | -15.0, 10.0 | -6.0, 3.0 |
| 2 | 0.976 (0.969 - 0.981) | 0.886 (0.854 - 0.912) | 0.911 (0.886 - 0.928) | 0.967 (0.959 - 0.973) |
|  | -7.3, 8.0 | -9.8, 16.2 | -16.0, 10.0 | -10.0, 4.0 |
| 3 | 0.959 (0.949 - 0.969) | 0.854 (0.807 - 0.885) | 0.897 (0.87 - 0.918) | 0.943 (0.933 - 0.952) |
|  | -9.0, 11.0 | -10.4, 17.3 | -13.0, 11.6 | -13.0, 5.0 |
| 4 | 0.943 (0.929 - 0.953) | 0.835 (0.787 - 0.863) | 0.88 (0.848 - 0.898) | 0.914 (0.894 - 0.929) |
|  | -10.6, 10.9 | -11.4, 16.9 | -13.0, 12.9 | -16.0, 8.0 |
| 5 | 0.928 (0.909 - 0.942) | 0.83 (0.783 - 0.858) | 0.867 (0.827 - 0.894) | 0.884 (0.85 - 0.906) |
|  | -13.1, 8.4 | -11.4, 15.6 | -12.5, 12.8 | -19.0, 7.0 |
| 6 | 0.909 (0.881 - 0.925) | 0.834 (0.791 - 0.87) | 0.852 (0.811 - 0.879) | 0.851 (0.809 - 0.879) |
|  | -13.1, 4.9 | -12.5, 16.0 | -13.6, 13.9 | -19.0, 7.0 |
| 7 | 0.885 (0.856 - 0.906) | 0.835 (0.776 - 0.866) | 0.831 (0.796 - 0.863) | 0.813 (0.775 - 0.85) |
|  | -15.7, 4.8 | -14.4, 16.3 | -15.3, 16.9 | -22.0, 8.0 |
| 8 | 0.849 (0.815 - 0.878) | 0.834 (0.785 - 0.881) | 0.805 (0.747 - 0.846) | 0.774 (0.725 - 0.819) |
|  | -18.5, 5.3 | -14.6, 15.9 | -19.5, 15.6 | -24.0, 7.0 |
| 9 | 0.805 (0.77 - 0.838) | 0.825 (0.777 - 0.864) | 0.775 (0.722 - 0.811) | 0.733 (0.684 - 0.779) |
|  | -22.3, 5.9 | -15.4, 14.0 | -22.3, 18.3 | -27.0, 5.0 |
| 10 | 0.756 (0.706 - 0.799) | 0.805 (0.759 - 0.838) | 0.746 (0.698 - 0.786) | 0.689 (0.618 - 0.744) |
|  | -24.6, 5.2 | -16.1, 12.4 | -23.6, 19.0 | -29.0, 5.0 |
| 11 | 0.698 (0.643 - 0.754) | 0.772 (0.714 - 0.81) | 0.718 (0.658 - 0.76) | 0.644 (0.571 - 0.703) |
|  | -26.0, 4.7 | -15.8, 11.3 | -24.6, 20.0 | -31.0, 6.0 |
| 12 | 0.62 (0.553 - 0.673) | 0.722 (0.652 - 0.781) | 0.686 (0.626 - 0.742) | 0.595 (0.518 - 0.677) |
|  | -29.3, 2.9 | -17.3, 10.8 | -24.5, 20.8 | -33.0, 6.0 |
| 13 | 0.531 (0.471 - 0.604) | 0.655 (0.572 - 0.708) | 0.647 (0.541 - 0.71) | 0.547 (0.471 - 0.628) |
|  | -35.0, 2.2 | -19.6, 10.2 | -29.5, 22.0 | -37.0, 8.0 |
| 14 | 0.443 (0.366 - 0.518) | 0.573 (0.494 - 0.637) | 0.61 (0.517 - 0.676) | 0.5 (0.415 - 0.574) |
|  | -38.7, 1.6 | -26.4, 9.0 | -32.5, 23.0 | -39.0, 7.0 |
| 15 | 0.344 (0.268 - 0.42) | 0.485 (0.404 - 0.544) | 0.567 (0.507 - 0.645) | 0.453 (0.37 - 0.54) |
|  | -41.7, 1.3 | -30.9, 7.1 | -33.5, 26.0 | -40.0, 7.0 |

**Supplementary Table 4:** *n*-day ventilator use forecasting results for the RF and baseline models in the monthly retrained setting. R^2^ and 95% CIs (top), and ME (bottom) were computed on the pooled predictions for the months after June 2020.

| **Mechanical ventilation** | | | | |
| --- | --- | --- | --- | --- |
| *n* | RF | LogR | LinR | Static |
| 1 | 0.973 (0.966 - 0.978) | 0.768 (0.684 - 0.824) | 0.863 (0.831 - 0.893) | 0.974 (0.967 - 0.98) |
|  | -3.7, 5.5 | -5.6, 14.6 | -11.8, 8.1 | -6.0, 3.0 |
| 2 | 0.936 (0.917 - 0.949) | 0.6 (0.47 - 0.704) | 0.853 (0.814 - 0.88) | 0.943 (0.928 - 0.955) |
|  | -4.1, 8.5 | -8.2, 19.6 | -11.8, 9.2 | -7.0, 4.0 |
| 3 | 0.903 (0.876 - 0.923) | 0.495 (0.39 - 0.615) | 0.844 (0.801 - 0.875) | 0.911 (0.893 - 0.927) |
|  | -5.0, 11.0 | -8.4, 22.3 | -10.8, 9.2 | -10.0, 4.0 |
| 4 | 0.878 (0.834 - 0.909) | 0.448 (0.337 - 0.568) | 0.826 (0.776 - 0.858) | 0.874 (0.841 - 0.897) |
|  | -6.6, 11.5 | -8.5, 22.5 | -9.8, 9.2 | -11.0, 5.0 |
| 5 | 0.854 (0.811 - 0.895) | 0.445 (0.324 - 0.54) | 0.801 (0.721 - 0.839) | 0.833 (0.792 - 0.863) |
|  | -7.8, 12.6 | -9.9, 20.9 | -9.8, 10.2 | -12.0, 4.0 |
| 6 | 0.841 (0.799 - 0.883) | 0.485 (0.379 - 0.562) | 0.769 (0.696 - 0.811) | 0.781 (0.744 - 0.822) |
|  | -8.9, 13.4 | -11.1, 19.2 | -10.7, 10.5 | -13.0, 5.0 |
| 7 | 0.839 (0.784 - 0.88) | 0.534 (0.466 - 0.594) | 0.737 (0.663 - 0.797) | 0.732 (0.684 - 0.784) |
|  | -9.0, 12.3 | -11.1, 17.6 | -13.7, 10.5 | -16.0, 6.0 |
| 8 | 0.83 (0.779 - 0.866) | 0.595 (0.508 - 0.655) | 0.699 (0.634 - 0.744) | 0.681 (0.616 - 0.73) |
|  | -9.3, 13.8 | -11.3, 16.0 | -14.1, 12.2 | -18.0, 6.0 |
| 9 | 0.811 (0.75 - 0.854) | 0.659 (0.6 - 0.707) | 0.661 (0.588 - 0.705) | 0.632 (0.574 - 0.689) |
|  | -10.7, 11.9 | -11.6, 14.1 | -15.3, 12.4 | -18.0, 4.0 |
| 10 | 0.784 (0.727 - 0.818) | 0.71 (0.645 - 0.758) | 0.608 (0.539 - 0.689) | 0.576 (0.521 - 0.646) |
|  | -12.1, 11.1 | -11.9, 10.6 | -15.3, 15.4 | -19.0, 4.0 |
| 11 | 0.74 (0.657 - 0.791) | 0.734 (0.638 - 0.786) | 0.551 (0.437 - 0.624) | 0.521 (0.421 - 0.59) |
|  | -14.5, 11.3 | -12.1, 10.4 | -16.4, 15.9 | -21.0, 5.0 |
| 12 | 0.685 (0.604 - 0.744) | 0.737 (0.664 - 0.794) | 0.504 (0.403 - 0.597) | 0.468 (0.365 - 0.555) |
|  | -16.9, 9.5 | -11.7, 9.7 | -19.4, 17.4 | -24.0, 4.0 |
| 13 | 0.619 (0.547 - 0.681) | 0.714 (0.647 - 0.772) | 0.452 (0.334 - 0.528) | 0.419 (0.322 - 0.5) |
|  | -18.9, 7.8 | -11.9, 8.0 | -18.4, 19.4 | -24.0, 5.0 |
| 14 | 0.537 (0.471 - 0.631) | 0.67 (0.579 - 0.722) | 0.403 (0.283 - 0.477) | 0.368 (0.248 - 0.502) |
|  | -21.5, 4.2 | -11.8, 7.1 | -18.5, 19.9 | -24.0, 5.0 |
| 15 | 0.446 (0.344 - 0.527) | 0.608 (0.511 - 0.68) | 0.358 (0.24 - 0.436) | 0.319 (0.203 - 0.432) |
|  | -24.0, 3.6 | -14.2, 5.9 | -22.4, 16.2 | -26.0, 5.0 |

**Supplementary Table 5:** *n*-day mortality forecasting results for the RF and the baseline logistic regression model in the monthly retrained setting (the LinR and static models do not provide predictions for mortality). R^2^ and 95% CIs (top), and ME (bottom) were computed on the pooled predictions for the months after June 2020.

| **Mortality** | | |
| --- | --- | --- |
| *n* | RF | LogR |
| 1 | 0.842 (0.794 - 0.877) | 0.676 (0.611 - 0.71) |
|  | -9.3, 9.6 | -13.5, 6.5 |
| 2 | 0.896 (0.851 - 0.922) | 0.718 (0.66 - 0.782) |
|  | -9.0, 15.8 | -23.8, 9.7 |
| 3 | 0.919 (0.887 - 0.939) | 0.73 (0.677 - 0.774) |
|  | -9.2, 20.0 | -36.9, 13.5 |
| 4 | 0.936 (0.918 - 0.952) | 0.736 (0.674 - 0.792) |
|  | -9.6, 24.5 | -45.6, 18.2 |
| 5 | 0.945 (0.928 - 0.957) | 0.736 (0.685 - 0.79) |
|  | -18.0, 30.9 | -54.6, 22.5 |
| 6 | 0.954 (0.946 - 0.964) | 0.737 (0.677 - 0.789) |
|  | -21.2, 27.7 | -61.9, 25.5 |
| 7 | 0.956 (0.944 - 0.967) | 0.735 (0.675 - 0.783) |
|  | -30.2, 25.4 | -69.2, 27.4 |
| 8 | 0.957 (0.946 - 0.967) | 0.73 (0.675 - 0.777) |
|  | -35.9, 26.4 | -79.5, 29.9 |
| 9 | 0.954 (0.941 - 0.968) | 0.722 (0.672 - 0.774) |
|  | -44.7, 22.5 | -94.6, 32.1 |
| 10 | 0.946 (0.931 - 0.964) | 0.712 (0.665 - 0.76) |
|  | -52.6, 15.4 | -107.8, 33.8 |
| 11 | 0.935 (0.91 - 0.955) | 0.7 (0.631 - 0.762) |
|  | -64.3, 9.1 | -117.3, 36.3 |
| 12 | 0.921 (0.892 - 0.942) | 0.685 (0.611 - 0.749) |
|  | -74.8, 9.7 | -126.0, 38.3 |
| 13 | 0.905 (0.874 - 0.928) | 0.669 (0.614 - 0.731) |
|  | -86.2, 9.8 | -133.4, 39.1 |
| 14 | 0.887 (0.856 - 0.912) | 0.651 (0.591 - 0.706) |
|  | -95.3, 9.9 | -146.5, 41.0 |
| 15 | 0.867 (0.829 - 0.902) | 0.633 (0.576 - 0.697) |
|  | -107.4, 8.1 | -153.0, 40.6 |

**Supplementary Table 6:** *n*-day hospital forecasting results for the RF and the baseline logistic regression model in the first wave setting. R^2^ and 95% CIs (top), and ME (bottom) were computed on the predictions for the months after June 2020.

| **Hospital** | | | | |
| --- | --- | --- | --- | --- |
| *n* | RF | LogR | LinR | Static |
| 1 | 0.985 (0.982 - 0.988) | 0.874 (0.812 - 0.912) | 0.85 (0.819 - 0.877) | 0.983 (0.976 - 0.987) |
|  | -77.7, 6.4 | -93.0, 225.6 | -168.2, 64.8 | -88.0, 20.0 |
| 2 | 0.95 (0.943 - 0.956) | 0.892 (0.856 - 0.916) | 0.847 (0.811 - 0.87) | 0.97 (0.963 - 0.979) |
|  | -125.7, 4.5 | -131.0, 200.6 | -195.9, 65.5 | -98.0, 36.0 |
| 3 | 0.909 (0.9 - 0.922) | 0.911 (0.89 - 0.931) | 0.844 (0.81 - 0.867) | 0.956 (0.943 - 0.968) |
|  | -155.2, 4.7 | -136.9, 151.5 | -193.6, 66.4 | -131.0, 54.0 |
| 4 | 0.854 (0.839 - 0.869) | 0.918 (0.899 - 0.932) | 0.844 (0.81 - 0.87) | 0.941 (0.925 - 0.953) |
|  | -189.6, 3.0 | -161.2, 96.1 | -182.6, 65.5 | -140.0, 58.0 |
| 5 | 0.79 (0.768 - 0.81) | 0.902 (0.88 - 0.924) | 0.846 (0.814 - 0.866) | 0.926 (0.9 - 0.948) |
|  | -209.2, 4.0 | -179.7, 48.8 | -151.6, 67.5 | -149.0, 74.0 |
| 6 | 0.717 (0.698 - 0.744) | 0.863 (0.842 - 0.885) | 0.847 (0.817 - 0.873) | 0.91 (0.881 - 0.932) |
|  | -239.5, 3.1 | -196.8, 42.4 | -159.6, 67.5 | -164.0, 66.0 |
| 7 | 0.632 (0.595 - 0.658) | 0.799 (0.766 - 0.83) | 0.842 (0.803 - 0.867) | 0.891 (0.865 - 0.92) |
|  | -282.7, 3.0 | -215.4, 32.8 | -173.5, 75.0 | -189.0, 75.0 |
| 8 | 0.542 (0.501 - 0.584) | 0.727 (0.699 - 0.757) | 0.829 (0.798 - 0.857) | 0.866 (0.836 - 0.892) |
|  | -315.7, 3.3 | -237.8, 24.2 | -196.8, 103.0 | -211.0, 93.0 |
| 9 | 0.444 (0.394 - 0.484) | 0.65 (0.614 - 0.678) | 0.812 (0.763 - 0.844) | 0.839 (0.796 - 0.882) |
|  | -344.3, 2.0 | -253.4, 15.2 | -209.8, 126.0 | -232.0, 109.0 |
| 10 | 0.349 (0.292 - 0.401) | 0.569 (0.529 - 0.604) | 0.795 (0.758 - 0.828) | 0.81 (0.77 - 0.857) |
|  | -367.5, 1.4 | -270.9, 6.3 | -228.8, 138.0 | -248.0, 119.0 |
| 11 | 0.262 (0.193 - 0.32) | 0.485 (0.435 - 0.525) | 0.78 (0.736 - 0.814) | 0.781 (0.726 - 0.827) |
|  | -389.0, 0.9 | -284.4, 2.8 | -231.8, 140.0 | -255.0, 132.0 |
| 12 | 0.171 (0.109 - 0.235) | 0.396 (0.352 - 0.435) | 0.767 (0.729 - 0.802) | 0.751 (0.697 - 0.827) |
|  | -410.4, 0.2 | -309.4, 2.2 | -227.0, 122.0 | -276.0, 142.0 |
| 13 | 0.09 (0.028 - 0.156) | 0.306 (0.24 - 0.356) | 0.754 (0.694 - 0.789) | 0.72 (0.661 - 0.795) |
|  | -428.8, 0.0 | -351.2, 1.6 | -271.0, 131.0 | -305.0, 139.0 |
| 14 | 0.009 (-0.049 - 0.062) | 0.219 (0.161 - 0.253) | 0.737 (0.683 - 0.775) | 0.688 (0.612 - 0.766) |
|  | -443.9, -0.3 | -378.8, 0.6 | -292.8, 140.0 | -320.0, 120.0 |
| 15 | -0.064 (-0.136 - -0.006) | 0.135 (0.065 - 0.194) | 0.712 (0.656 - 0.761) | 0.653 (0.583 - 0.724) |
|  | -459.5, -0.9 | -401.4, 0.1 | -309.0, 143.0 | -348.0, 127.0 |

**Supplementary Table 7:** *n*-day ICU admission forecasting results for the RF and the baseline logistic regression model in the first wave setting. R^2^ and 95% CIs (top), and ME (bottom) were computed on the predictions for the months after June 2020.

| **ICU admission** | | | | |
| --- | --- | --- | --- | --- |
| *n* | RF | LogR | LinR | Static |
| 1 | 0.988 (0.985 - 0.991) | 0.729 (0.64 - 0.781) | 0.843 (0.804 - 0.873) | 0.986 (0.983 - 0.989) |
|  | -5.0, 4.9 | -7.4, 30.5 | -15.2, 13.4 | -6.0, 3.0 |
| 2 | 0.972 (0.965 - 0.977) | 0.488 (0.348 - 0.614) | 0.835 (0.793 - 0.868) | 0.967 (0.96 - 0.973) |
|  | -6.6, 8.5 | -7.5, 39.9 | -16.2, 12.5 | -10.0, 4.0 |
| 3 | 0.954 (0.945 - 0.964) | 0.293 (0.104 - 0.454) | 0.827 (0.794 - 0.857) | 0.943 (0.93 - 0.955) |
|  | -6.8, 12.1 | -7.6, 46.5 | -13.5, 12.6 | -13.0, 5.0 |
| 4 | 0.942 (0.93 - 0.953) | 0.183 (-0.048 - 0.374) | 0.814 (0.773 - 0.84) | 0.914 (0.894 - 0.932) |
|  | -8.0, 10.7 | -8.3, 49.4 | -13.2, 16.6 | -16.0, 8.0 |
| 5 | 0.94 (0.922 - 0.95) | 0.166 (-0.026 - 0.341) | 0.805 (0.748 - 0.839) | 0.884 (0.858 - 0.905) |
|  | -9.2, 10.6 | -8.4, 47.7 | -10.5, 15.8 | -19.0, 7.0 |
| 6 | 0.93 (0.913 - 0.943) | 0.229 (0.074 - 0.373) | 0.795 (0.747 - 0.832) | 0.851 (0.817 - 0.875) |
|  | -9.1, 9.5 | -9.0, 48.2 | -11.3, 17.6 | -19.0, 7.0 |
| 7 | 0.915 (0.891 - 0.929) | 0.31 (0.19 - 0.429) | 0.777 (0.706 - 0.816) | 0.813 (0.777 - 0.852) |
|  | -11.1, 10.3 | -10.4, 46.2 | -12.7, 20.6 | -22.0, 8.0 |
| 8 | 0.896 (0.872 - 0.916) | 0.413 (0.293 - 0.488) | 0.755 (0.7 - 0.802) | 0.774 (0.728 - 0.818) |
|  | -13.7, 10.2 | -11.4, 43.3 | -16.7, 19.8 | -24.0, 7.0 |
| 9 | 0.873 (0.837 - 0.893) | 0.529 (0.449 - 0.604) | 0.732 (0.664 - 0.771) | 0.733 (0.683 - 0.777) |
|  | -17.2, 10.3 | -12.3, 38.8 | -19.7, 18.6 | -27.0, 5.0 |
| 10 | 0.851 (0.815 - 0.876) | 0.637 (0.582 - 0.692) | 0.708 (0.656 - 0.751) | 0.689 (0.63 - 0.75) |
|  | -20.1, 10.5 | -13.2, 33.7 | -20.7, 20.3 | -29.0, 5.0 |
| 11 | 0.814 (0.762 - 0.852) | 0.724 (0.665 - 0.762) | 0.687 (0.611 - 0.734) | 0.644 (0.569 - 0.709) |
|  | -22.8, 9.7 | -13.1, 31.9 | -21.6, 21.3 | -31.0, 6.0 |
| 12 | 0.751 (0.702 - 0.793) | 0.79 (0.752 - 0.824) | 0.66 (0.597 - 0.73) | 0.595 (0.525 - 0.669) |
|  | -26.0, 7.1 | -14.6, 26.8 | -21.5, 21.3 | -33.0, 6.0 |
| 13 | 0.69 (0.624 - 0.75) | 0.828 (0.797 - 0.859) | 0.626 (0.546 - 0.696) | 0.547 (0.442 - 0.623) |
|  | -29.0, 6.5 | -14.8, 19.5 | -26.5, 23.6 | -37.0, 8.0 |
| 14 | 0.601 (0.516 - 0.668) | 0.828 (0.782 - 0.86) | 0.593 (0.489 - 0.671) | 0.5 (0.406 - 0.592) |
|  | -34.1, 5.1 | -15.2, 13.9 | -29.5, 24.3 | -39.0, 7.0 |
| 15 | 0.509 (0.439 - 0.609) | 0.797 (0.756 - 0.831) | 0.553 (0.469 - 0.628) | 0.453 (0.353 - 0.524) |
|  | -37.6, 3.0 | -19.1, 11.5 | -30.5, 27.3 | -40.0, 7.0 |

**Supplementary Table 8:** *n*-day ventilator use forecasting results for the RF and the baseline logistic regression model in the first wave setting. R^2^ and 95% CIs (top), and ME (bottom) were computed on the predictions for the months after June 2020.

| **Mechanical ventilation** | | | | |
| --- | --- | --- | --- | --- |
| n | RF | LogR | LinR | Static |
| 1 | 0.922 (0.888 - 0.945) | -0.05 (-0.412 - 0.131) | 0.725 (0.643 - 0.765) | 0.974 (0.966 - 0.98) |
|  | -2.6, 10.4 | -3.9, 31.4 | -10.5, 9.8 | -6.0, 3.0 |
| 2 | 0.817 (0.767 - 0.863) | -0.935 (-1.467 - -0.637) | 0.72 (0.665 - 0.78) | 0.943 (0.933 - 0.959) |
|  | -3.5, 15.7 | -6.0, 43.4 | -10.5, 9.8 | -7.0, 4.0 |
| 3 | 0.7 (0.611 - 0.78) | -1.535 (-2.166 - -1.05) | 0.715 (0.657 - 0.772) | 0.911 (0.888 - 0.929) |
|  | -3.9, 18.6 | -5.6, 50.2 | -9.5, 9.2 | -10.0, 4.0 |
| 4 | 0.664 (0.559 - 0.732) | -1.857 (-2.534 - -1.302) | 0.702 (0.629 - 0.757) | 0.874 (0.84 - 0.898) |
|  | -5.0, 19.3 | -5.4, 52.7 | -9.2, 9.2 | -11.0, 5.0 |
| 5 | 0.649 (0.558 - 0.733) | -1.927 (-2.486 - -1.39) | 0.678 (0.555 - 0.732) | 0.833 (0.798 - 0.86) |
|  | -6.1, 20.1 | -6.7, 51.3 | -9.2, 11.0 | -12.0, 4.0 |
| 6 | 0.672 (0.576 - 0.76) | -1.785 (-2.367 - -1.253) | 0.65 (0.592 - 0.715) | 0.781 (0.736 - 0.823) |
|  | -7.4, 19.2 | -8.2, 50.7 | -10.1, 13.3 | -13.0, 5.0 |
| 7 | 0.68 (0.56 - 0.764) | -1.552 (-2.077 - -1.096) | 0.621 (0.536 - 0.697) | 0.732 (0.683 - 0.785) |
|  | -7.5, 18.3 | -8.1, 48.8 | -11.7, 13.3 | -16.0, 6.0 |
| 8 | 0.705 (0.613 - 0.774) | -1.213 (-1.612 - -0.905) | 0.587 (0.481 - 0.651) | 0.681 (0.62 - 0.727) |
|  | -8.1, 18.9 | -8.2, 46.3 | -12.6, 13.9 | -18.0, 6.0 |
| 9 | 0.731 (0.657 - 0.8) | -0.815 (-1.14 - -0.578) | 0.554 (0.463 - 0.631) | 0.632 (0.565 - 0.695) |
|  | -9.2, 16.9 | -8.9, 42.0 | -13.4, 13.2 | -18.0, 4.0 |
| 10 | 0.747 (0.685 - 0.787) | -0.422 (-0.608 - -0.231) | 0.506 (0.38 - 0.608) | 0.576 (0.498 - 0.645) |
|  | -10.7, 14.8 | -9.4, 36.9 | -13.4, 16.2 | -19.0, 4.0 |
| 11 | 0.733 (0.648 - 0.784) | -0.107 (-0.266 - 0.081) | 0.455 (0.306 - 0.549) | 0.521 (0.42 - 0.603) |
|  | -12.7, 14.8 | -9.8, 32.6 | -13.1, 16.1 | -21.0, 5.0 |
| 12 | 0.718 (0.643 - 0.764) | 0.146 (0.009 - 0.323) | 0.413 (0.291 - 0.509) | 0.468 (0.391 - 0.536) |
|  | -14.7, 13.9 | -9.5, 28.7 | -16.1, 18.2 | -24.0, 4.0 |
| 13 | 0.692 (0.636 - 0.754) | 0.353 (0.182 - 0.466) | 0.364 (0.234 - 0.485) | 0.419 (0.324 - 0.517) |
|  | -16.0, 12.3 | -9.5, 25.6 | -15.1, 20.2 | -24.0, 5.0 |
| 14 | 0.645 (0.584 - 0.704) | 0.496 (0.38 - 0.645) | 0.318 (0.192 - 0.414) | 0.368 (0.274 - 0.473) |
|  | -19.0, 8.7 | -9.6, 24.6 | -16.7, 20.1 | -24.0, 5.0 |
| 15 | 0.576 (0.487 - 0.658) | 0.586 (0.482 - 0.695) | 0.275 (0.133 - 0.396) | 0.319 (0.197 - 0.435) |
|  | -21.5, 8.1 | -10.0, 22.9 | -19.1, 17.9 | -26.0, 5.0 |

**Supplementary Table 9:** *n*-day mortality forecasting results for the RF and the baseline logistic regression model in the first wave setting (the LinR and static models do not provide predictions for mortality). R^2^ and 95% CIs (top), and ME (bottom) were computed on the predictions for the months after June 2020.

| **Mortality** | | |
| --- | --- | --- |
| *n* | RF | LogR |
| 1 | 0.581 (0.411 - 0.713) | 0.373 (0.086 - 0.537) |
|  | -5.3, 14.6 | -9.0, 17.1 |
| 2 | 0.644 (0.545 - 0.74) | 0.419 (0.275 - 0.519) |
|  | -3.8, 25.6 | -15.6, 27.6 |
| 3 | 0.679 (0.587 - 0.747) | 0.463 (0.273 - 0.581) |
|  | -5.3, 35.8 | -25.2, 39.1 |
| 4 | 0.708 (0.647 - 0.78) | 0.504 (0.378 - 0.593) |
|  | -5.2, 46.8 | -29.9, 49.6 |
| 5 | 0.725 (0.651 - 0.787) | 0.546 (0.452 - 0.636) |
|  | -5.6, 60.2 | -35.6, 58.0 |
| 6 | 0.773 (0.719 - 0.816) | 0.592 (0.517 - 0.661) |
|  | -6.2, 62.3 | -40.2, 64.0 |
| 7 | 0.811 (0.766 - 0.843) | 0.636 (0.578 - 0.711) |
|  | -10.0, 63.0 | -44.7, 69.5 |
| 8 | 0.853 (0.82 - 0.884) | 0.677 (0.606 - 0.738) |
|  | -14.8, 66.5 | -50.3, 73.5 |
| 9 | 0.884 (0.849 - 0.909) | 0.718 (0.677 - 0.772) |
|  | -23.7, 69.3 | -58.1, 72.8 |
| 10 | 0.914 (0.901 - 0.933) | 0.755 (0.716 - 0.793) |
|  | -26.5, 63.1 | -67.0, 71.6 |
| 11 | 0.929 (0.916 - 0.941) | 0.786 (0.75 - 0.814) |
|  | -36.0, 52.7 | -75.1, 66.1 |
| 12 | 0.937 (0.925 - 0.946) | 0.809 (0.759 - 0.836) |
|  | -47.7, 49.0 | -82.0, 63.5 |
| 13 | 0.941 (0.927 - 0.951) | 0.825 (0.795 - 0.848) |
|  | -60.6, 38.7 | -87.7, 61.7 |
| 14 | 0.938 (0.922 - 0.953) | 0.835 (0.809 - 0.866) |
|  | -69.3, 34.7 | -99.1, 57.1 |
| 15 | 0.931 (0.913 - 0.951) | 0.841 (0.806 - 0.868) |
|  | -78.5, 27.3 | -103.8, 49.9 |

**Supplementary Table 10:** ROC-AUC score and 95% confidence intervals for the RF and LogR models used for making *n*-day individual predictions in the monthly retrained setting. For every target, RF was found to perform significantly better than LogR when tested with the DeLong test (*p* < 0.0001).

|  | **RF** | | | | **LogR** | | | |
| --- | --- | --- | --- | --- | --- | --- | --- | --- |
| *n* | Hospital admission | ICU admission | Mechanical ventilation | Mortality | Hospital admission | ICU admission | Mechanical ventilation | Mortality |
| 1 | 0.992 | 0.995 | 0.997 | 0.914 | 0.953 | 0.97 | 0.977 | 0.772 |
|  | (0.991 - 0.992) | (0.993 - 0.997) | (0.996 - 0.998) | (0.901 - 0.922) | (0.951 - 0.955) | (0.966 - 0.974) | (0.972 - 0.982) | (0.758 - 0.784) |
| 2 | 0.985 | 0.995 | 0.997 | 0.924 | 0.937 | 0.956 | 0.963 | 0.771 |
|  | (0.983 - 0.985) | (0.994 - 0.996) | (0.996 - 0.998) | (0.918 - 0.929) | (0.935 - 0.94) | (0.95 - 0.96) | (0.957 - 0.969) | (0.763 - 0.78) |
| 3 | 0.978 | 0.992 | 0.994 | 0.941 | 0.922 | 0.947 | 0.956 | 0.764 |
|  | (0.977 - 0.98) | (0.989 - 0.994) | (0.991 - 0.996) | (0.936 - 0.946) | (0.92 - 0.925) | (0.941 - 0.954) | (0.948 - 0.962) | (0.756 - 0.771) |
| 4 | 0.973 | 0.991 | 0.994 | 0.947 | 0.914 | 0.931 | 0.938 | 0.767 |
|  | (0.971 - 0.974) | (0.988 - 0.993) | (0.991 - 0.996) | (0.944 - 0.95) | (0.911 - 0.918) | (0.924 - 0.937) | (0.928 - 0.945) | (0.761 - 0.774) |
| 5 | 0.968 | 0.99 | 0.994 | 0.952 | 0.904 | 0.926 | 0.933 | 0.763 |
|  | (0.966 - 0.97) | (0.986 - 0.992) | (0.991 - 0.996) | (0.949 - 0.955) | (0.9 - 0.908) | (0.918 - 0.933) | (0.923 - 0.943) | (0.757 - 0.769) |
| 6 | 0.965 | 0.992 | 0.995 | 0.952 | 0.898 | 0.923 | 0.928 | 0.762 |
|  | (0.963 - 0.967) | (0.991 - 0.993) | (0.994 - 0.995) | (0.949 - 0.956) | (0.895 - 0.901) | (0.916 - 0.929) | (0.919 - 0.938) | (0.755 - 0.769) |
| 7 | 0.96 | 0.989 | 0.991 | 0.954 | 0.888 | 0.904 | 0.906 | 0.757 |
|  | (0.958 - 0.962) | (0.987 - 0.991) | (0.989 - 0.993) | (0.952 - 0.956) | (0.885 - 0.893) | (0.894 - 0.913) | (0.895 - 0.917) | (0.751 - 0.763) |
| 8 | 0.956 | 0.989 | 0.989 | 0.953 | 0.88 | 0.901 | 0.907 | 0.753 |
|  | (0.954 - 0.959) | (0.987 - 0.991) | (0.985 - 0.992) | (0.951 - 0.956) | (0.875 - 0.884) | (0.893 - 0.909) | (0.897 - 0.917) | (0.747 - 0.759) |
| 9 | 0.958 | 0.985 | 0.988 | 0.954 | 0.883 | 0.892 | 0.898 | 0.744 |
|  | (0.955 - 0.96) | (0.982 - 0.988) | (0.985 - 0.991) | (0.952 - 0.957) | (0.879 - 0.889) | (0.883 - 0.901) | (0.886 - 0.91) | (0.736 - 0.749) |
| 10 | 0.959 | 0.986 | 0.986 | 0.954 | 0.876 | 0.881 | 0.889 | 0.738 |
|  | (0.956 - 0.961) | (0.982 - 0.989) | (0.981 - 0.99) | (0.952 - 0.957) | (0.872 - 0.881) | (0.872 - 0.893) | (0.874 - 0.9) | (0.731 - 0.744) |
| 11 | 0.958 | 0.985 | 0.987 | 0.955 | 0.873 | 0.866 | 0.871 | 0.732 |
|  | (0.956 - 0.961) | (0.982 - 0.987) | (0.981 - 0.991) | (0.952 - 0.956) | (0.865 - 0.879) | (0.854 - 0.876) | (0.855 - 0.88) | (0.728 - 0.739) |
| 12 | 0.96 | 0.984 | 0.985 | 0.955 | 0.874 | 0.867 | 0.864 | 0.722 |
|  | (0.957 - 0.962) | (0.981 - 0.987) | (0.979 - 0.989) | (0.953 - 0.957) | (0.869 - 0.879) | (0.854 - 0.878) | (0.847 - 0.881) | (0.713 - 0.728) |
| 13 | 0.956 | 0.984 | 0.983 | 0.954 | 0.859 | 0.856 | 0.861 | 0.706 |
|  | (0.953 - 0.959) | (0.979 - 0.986) | (0.975 - 0.987) | (0.952 - 0.956) | (0.852 - 0.866) | (0.843 - 0.87) | (0.846 - 0.88) | (0.7 - 0.714) |
| 14 | 0.959 | 0.985 | 0.985 | 0.955 | 0.856 | 0.848 | 0.854 | 0.7 |
|  | (0.957 - 0.962) | (0.981 - 0.988) | (0.98 - 0.99) | (0.953 - 0.957) | (0.849 - 0.863) | (0.832 - 0.86) | (0.84 - 0.87) | (0.692 - 0.705) |
| 15 | 0.956 | 0.981 | 0.982 | 0.953 | 0.847 | 0.838 | 0.846 | 0.686 |
|  | (0.953 - 0.96) | (0.976 - 0.984) | (0.974 - 0.987) | (0.951 - 0.955) | (0.839 - 0.855) | (0.827 - 0.852) | (0.832 - 0.86) | (0.678 - 0.693) |
